# A minimally invasive, scalable and reproducible neonatal rat model of severe focal brain injury

**DOI:** 10.1093/braincomms/fcag171

**Published:** 2026-05-15

**Authors:** Victor Mondal, Emily Ross-Munro, Gayathri K Balasuriya, Ritu Kumari, Isabelle K Shearer, Andjela Micic, Abdullah Al Mamun Sohag, Alan Shi, Mikaela Barresi, David R Nisbet, Glenn F King, Richard J Williams, Pierre Gressens, Flora Y Wong, Jeanie L Y Cheong, David W Walker, Mary Tolcos, Bobbi Fleiss

**Affiliations:** School of Health and Biomedical Sciences, RMIT University, Bundoora, Melbourne, Victoria 3083, Australia; School of Health and Biomedical Sciences, RMIT University, Bundoora, Melbourne, Victoria 3083, Australia; School of Health and Biomedical Sciences, RMIT University, Bundoora, Melbourne, Victoria 3083, Australia; School of Health and Biomedical Sciences, RMIT University, Bundoora, Melbourne, Victoria 3083, Australia; School of Health and Biomedical Sciences, RMIT University, Bundoora, Melbourne, Victoria 3083, Australia; School of Health and Biomedical Sciences, RMIT University, Bundoora, Melbourne, Victoria 3083, Australia; School of Health and Biomedical Sciences, RMIT University, Bundoora, Melbourne, Victoria 3083, Australia; School of Health and Biomedical Sciences, RMIT University, Bundoora, Melbourne, Victoria 3083, Australia; School of Health and Biomedical Sciences, RMIT University, Bundoora, Melbourne, Victoria 3083, Australia; Aikenhead Centre for Medical Discovery, St Vincent's Hospital, Fitzroy, Melbourne, Victoria 3065, Australia; Department of Biomedical Engineering, Faculty of Engineering and Information Technology, The University of Melbourne, Parkville, Melbourne, Victoria 3010, Australia; The Graeme Clark Institute, The University of Melbourne, Parkville, Melbourne, Victoria 3010, Australia; Medical School, Faculty of Medicine, Dentistry and Health Science, The University of Melbourne, Parkville, Melbourne, Victoria 3010, Australia; Institute for Molecular Bioscience, The University of Queensland, St Lucia, Queensland 4072, Australia; Australian Research Council Centre of Excellence for Innovations in Peptide and Protein Science, The University of Queensland, St Lucia, Queensland 4072, Australia; School of Health and Biomedical Sciences, RMIT University, Bundoora, Melbourne, Victoria 3083, Australia; The Graeme Clark Institute, The University of Melbourne, Parkville, Melbourne, Victoria 3010, Australia; School of Medicine, Deakin University, Waurn Ponds, Geelong, Victoria 3216, Australia; Université Paris Cité, Inserm, NeuroDiderot, Paris 75019, France; Monash Newborn, Monash Children’s Hospital and Department of Paediatrics, Monash University, Melbourne, Victoria 3168, Australia; The Ritchie Centre, Hudson Institute of Medical Research, Melbourne, Victoria 3168, Australia; Clinical Sciences, Murdoch Children’s Research Institute, Parkville, Melbourne, Victoria 3052, Australia; Neonatal Services, Royal Women’s Hospital, Parkville, Melbourne, Victoria 3052, Australia; Department of Obstetrics, Gynaecology and Newborn Health, and Department of Paediatrics, University of Melbourne, Parkville, Melbourne, Victoria 3052, Australia; School of Health and Biomedical Sciences, RMIT University, Bundoora, Melbourne, Victoria 3083, Australia; The Ritchie Centre, Hudson Institute of Medical Research, Melbourne, Victoria 3168, Australia; School of Health and Biomedical Sciences, RMIT University, Bundoora, Melbourne, Victoria 3083, Australia; School of Health and Biomedical Sciences, RMIT University, Bundoora, Melbourne, Victoria 3083, Australia; Université Paris Cité, Inserm, NeuroDiderot, Paris 75019, France

**Keywords:** stroke, thrombosis, ischaemia, neuroinflammation, perinatal

## Abstract

Neonatal brain injuries, such as stroke, cause focal ischaemic lesions that often result in lifelong neurological disabilities, yet effective treatments remain limited. Early-phase therapeutic screening requires models that can reliably reproduce injury severity while minimising confounding variables, including prolonged or variable anaesthesia, surgical stress, and invasive procedures that themselves affect injury progression. Existing models of neonatal focal ischaemia often exhibit high mortality, technical complexity, and substantial variability in lesion location and volume. As a result, there is a critical need for a rapid, ethically refined, and scalable neonatal model that produces consistent cortical injury suitable for screening neuroprotective, biomaterial-based, and regenerative therapies. We established a minimally invasive photothrombotic ischaemia model in postnatal day 10 rats by administering intraperitoneal Rose Bengal (25, 40, or 60 mg/kg) and activating it with a fixed 10-minute exposure to 565-nm light through the intact scalp and skull. This incision-free protocol allowed a total procedure duration of 19 min. We characterized dose-dependent effects on infarct volume and anatomical distribution, cortical atrophy, ventricular enlargement, apoptosis (cleaved caspase-3), astrocytic and microglial reactivity (glial fibrillary acidic protein, GFAP; ionized calcium-binding adapter molecule 1, Iba1), and sensorimotor outcomes (wire hang, cylinder rearing, adhesive tape removal) at 1, 7, and 14 days after injury. Additional analyses assessed the reproducibility of lesion size across litters and explored sex-specific differences. A 25 mg/kg dose induced a reliable and well-localized motor cortex infarct with no mortality. Higher doses of Rose Bengal produced proportionally larger infarcts with greater subcortical involvement and more pronounced secondary atrophy. Across all groups, apoptotic signalling and glial reactivity remained elevated through 14 days, indicating persistent tissue injury. Sensorimotor impairments were robust at all stages, with deficits in forepaw use, endurance, and tactile response correlating with lesion volumes in the 25 mg/kg group. No significant sex differences were observed for any histological or behavioural outcomes. This refined neonatal photothrombotic model provides a reproducible, simple, scalable, and ethically optimized platform for inducing severe focal cortical injury. The model’s stable injury territory, short, standardized procedure, and consistent functional readouts fill a major gap in current research tools and provide a practical foundation for early-phase testing of neuroprotective and regenerative interventions.

## Teaser

Mondal *et al*. establish a minimally invasive photothrombotic model in term-equivalent neonatal rats that produces reproducible, dose-dependent cortical infarcts with persistent gliosis and sensorimotor deficits. This scalable, ethically refined approach offers a robust platform for early-phase screening of neuroprotective and regenerative therapies.

## Graphical Abstract

**Graphical Abstract:**
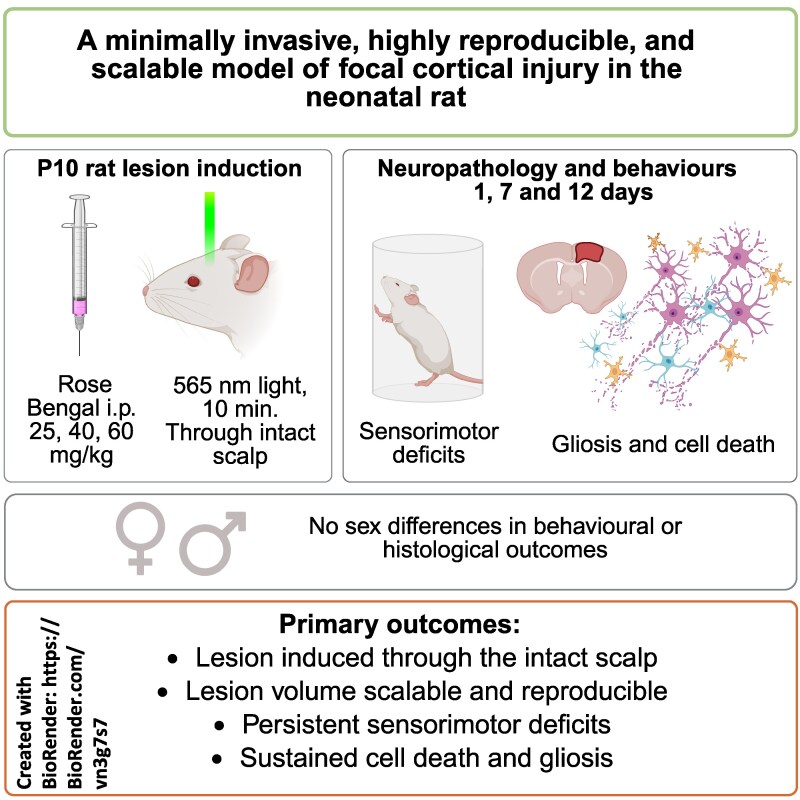

## Introduction

Injury to the developing brain is the most common cause of permanent disability worldwide.^1^ Infants with the most severe focal brain injury, particularly those linked to focal ischaemia, are at high risk of significant lifelong disability. For instance, most infants who suffer a stroke in the region of the middle cerebral artery in the first 28 days of their postnatal lives develop cerebral palsy, and over 80% will exhibit language, hearing, or visual field impairments.^2,3^ Approximately 1 in 2500 to 7500 infants suffer from an arterial stroke in the first 30 days of life, and these frequently lead to injury to the lateral frontal, parietal, and temporal cortices.^4^ In addition, following severe hypoxic-ischaemic encephalopathy (HIE), 25% of infants develop cerebral palsy, and 70% of those without a motor deficit will experience significant academic challenges.^5^ Only 10–15% of cases of HIE are severe, but 40% of these are characterized as occurring due to prolonged partial asphyxia, which is characterized by watershed and cortical injury.^6,7^ Among children under one year of age, accidental or abusive head trauma is the leading cause of traumatic brain injury (TBI)-related death and hospitalisation.^8,9^ It is estimated that globally, 600 per 100 000 children under 16 will suffer a TBI each year, with the rates highest in the first 12 months of life.^10-12^ These TBIs are often associated with large, focal lesions on clinical imaging, including common cerebral contusion,^13,14^ and more than 70% of infants with severe TBI who survive live with adverse cognitive and motor outcomes.^15^

Traditionally, HIE and TBI are considered diffuse injuries, but as outlined above in their most severe presentations, both involve focal cortical or subcortical infarctions, which are linked to ischaemia.^16^ Currently, there are no curative therapies for severe focal brain injuries. Therapeutic hypothermia, the current standard of care for HIE, is only partially effective, has limited applicability to other types of brain injuries^17,18^ and is effective only in a resource-intensive setting.^19-21^ Severe brain injury is dramatically more prevalent in regions where there are inequalities in access to health care.^19^ This highlights the need for preclinical models that reproduce focal cortical lesions in the neocortex to support the development of therapies, including first-line screening models. Addressing this need requires models that are reproducible, ethically refined, and scalable across different injury severities, especially for early-phase therapy screening.^22^

Whilst several models exist to study focal brain injury, these have limitations for early-phase screening, especially when fixed scalability is required. The middle cerebral artery occlusion (MCAO) model is widely used for its clinical relevance, but infarct size varies widely across animals due to differences in collateral blood flow,^23^ and mortality can reach 40%.^24,25^ This limits reliable scalability and demands large group sizes. The endothelin-1 model has lower mortality but still shows inconsistent lesion size.^26^ The Rice-Vannucci model, commonly used for HIE, is similarly limited by variable lesion territories despite lower mortality (5–10%).^27^ All three models are invasive, technically complex, and poorly suited for high-throughput early-phase neurotherapy screening.

In contrast, the photothrombosis model has a low mortality rate (<2%) and a highly reproducible lesion territory.^28-30^ Rose Bengal, a visible-light photoredox catalyst, is administered systemically, followed by targeted light exposure onto the brain to induce platelet aggregation within cerebral blood vessels, resulting in focal vascular occlusion. In the adult, the scalp, skin, periosteum, and skull cause scattering and attenuation of light that necessitate at least an incision to apply the light directly to the skull,^28^ or on the brain following a craniotomy.^30^ The first iteration of this model in the neonatal rat required an injection of Rose Bengal into the exposed jugular vein and the application of light directly to the skull over the cortex after a skin incision.^31^ Since that time, there have been substantial improvements to reduce invasiveness, including intraperitoneal injection of Rose Bengal and only a skin incision for light placement.^32^

To the best of our knowledge, no minimally invasive protocol (Rose Bengal, intraperitoneal, no skin incision) with a fixed procedure duration has been established that correlates the Rose Bengal dose with highly reproducible infarct size and subsequent structural and functional damage. This is a critical gap, particularly for early-phase therapy development, where reproducibility and ethical refinement are essential, and for testing therapies such as hydrogel-based delivery systems and tissue transplants, where treatments are tailored to specific lesion characteristics.^4,33^ Moreover, standardising surgical duration is important for comparing injury severity, especially as anaesthesia itself increases cell death in the neonatal brain.^34^

Here, we established a minimally invasive, fixed-duration, scalable and highly reproducible method for inducing photothrombotic focal brain injury in neonatal rats, and characterized the temporal progression of glial activation, cell death, and associated behavioural deficits. We hypothesized that higher doses of Rose Bengal would lead to more severe structural and functional outcomes. We examined sex differences in injury outcomes, given that male infants are known to have worse neurological outcomes than females.^35^

## Materials and methods

### Animals and surgical procedures

Please also refer to the Supplementary Methods for further information. All experiments were approved by the RMIT University Animal Ethics Committee (AEC 1918) and are reported in accordance with the ARRIVE guidelines. Pregnant Wistar rats were obtained from Australian Bioresources (Perth, Western Australia) and allowed to deliver undisturbed. Pups were weighed on postnatal day (P) 8 and randomly assigned to treatment groups within litters using a restricted block design. P10 was chosen for the induction of focal cortical injury as it is developmentally equivalent to a term-born human infant.^36^

Predetermined exclusion criteria included humane endpoints and technical artefacts; only one pup (sham group) was excluded because it died before its endpoint. For histology, one pup per litter per timepoint was analysed; for behavioural assays, each pup was treated as an independent replicate. Sex differences were assessed by adding additional animals from independent litters to the 25 mg/kg Rose Bengal group. Across the study, the female: male pup ratio was 28:26.

P10 rats were anaesthetized with 5% isoflurane in 0.4 L/min O_2_ (2–3% maintenance) and kept normothermic on a thermistor-controlled pad (Thorlabs, USA). Rose Bengal (Merck, Australia; cat#330000) was injected intraperitoneally at 25, 40, or 60 mg/kg, based on a review of the literature.^30^ After 5 min, a 1 mm light-emitting diode (LED) light source (Thorlabs M565F3, 565 nm) was positioned via the arm of the stereotactic unit, 2 mm right of Bregma and illuminated through the intact scalp and skull for 10 min (Supplementary Fig. 1). The full procedure averaged 19 min for all groups.

**Figure 1:**
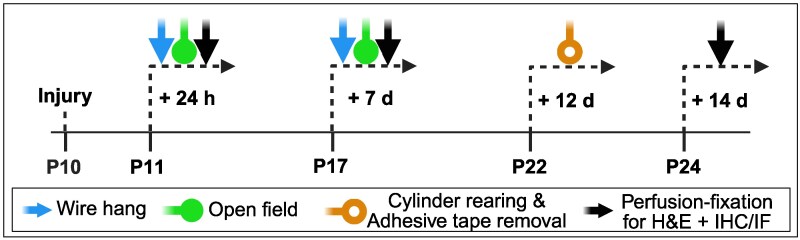
**Experimental design outline.** +, plus; d, day; h, hour; H&E, haematoxylin and eosin; IF, immunofluorescence; IHC, immunohistochemistry. Created in BioRender. Mondal, V. (2026) https://BioRender.com/1uwxbo5.

At completion, buprenorphine (0.05 mg/kg) and meloxicam (5 mg/kg) were administered subcutaneously. Pups recovered for 1 h on a heating pad before being returned to the dam. Sham controls received Rose Bengal without illumination to control for dye effects.^37^ Light exposure alone produces no detectable lesion and was not considered a confound. Further procedural details and anatomical schematics appear in Supplementary Methods and Fig. 1.

### Behavioural testing

Behavioural testing was conducted between 0800 and 1200 h by blinded assessors. Testing epochs corresponded to: P11 (24 h post-injury)—acute neuroinflammatory phase; P17 (7 days)—subacute consolidation/remodelling; P22–P24 (12–14 days)—early recovery, coinciding with rapid myelination and circuit maturation, approximating 2–3 years human age.^36,38,39^

#### Wire hang test

At P11 and P17, rats grasped a wire using both forepaws and were allowed to hang for ≤5 min. Three trials (30 s apart) were video-recorded, and the total hang time was summed.

#### Cylinder rearing test

At P22 (12 days post-injury), rats were filmed for 5 min in a transparent cylinder (185 mm height × 130 mm diameter). Forepaw contacts were classified as contralateral, ipsilateral, or bilateral, and contralateral use (%) = (contralateral touches/(contralateral + ipsilateral touches) × 100.

#### Adhesive-tape removal test

At P22, adhesive discs (0.95 cm diameter; Merck Z743588) were applied to both forepaws. Each rat completed three trials (maximum 3 min per trial, 1-minute interval). The time to contact and remove each tape was recorded.

#### Open-field test

At P11 and P17, rats explored a 30 × 30 cm arena for 3 min under overhead video. Parameters included locomotor activity, centre time, rearing, and immobility.

All behavioural paradigms are further detailed in Supplementary Methods and schematized in Fig. 1.

### Brain tissue collection and processing

Brains were collected at P11, P17, and P24 (Fig. 1). Animals were anaesthetized with Lethabarb (>200 mg/kg, Virbac Australia) + lignocaine (10 mg/mL, Cenvet, Australia) and perfused with PBS followed by 4% paraformaldehyde (PFA). Brains were post-fixed for 12–18 h, transferred to 70% ethanol, and processed (Leica ASP300S) into paraffin. Coronal sections (8 µm) were cut from Bregma +3.24 mm to beyond the lesion. Three sections per slide were mounted on Superfrost Plus slides (each section spaced 160 µm apart) and stored for analysis.

### Histological analyses

#### Haematoxylin and eosin (H&E)

Every 20th section was stained to define lesion morphology. Area and depth were measured in ImageJ as described previously.^40,41^ Infarct volume was estimated using Cavalieri’s principle (V = ΣA × P × T). Lesion location was referenced to the Paxinos and Watson atlas.^42^

#### Reproducibility and cortical atrophy

Lesion consistency between litters was quantified as the coefficient of variation (CV).^43^ Cortical atrophy = [1 – (ipsilateral/contralateral area)] × 100, averaged across sections, as previously reported.^41,44^ Ventricular enlargement was expressed as the lateral ventricular ratio (LVR).

### Immunohistochemistry (IHC) and immunofluorescence (IF)

IHC and IF were performed as previously described,^45,46^ with full details in Supplementary Methods. Sections were dewaxed, subjected to citrate buffer antigen retrieval (pH 6.0), and blocked in 5% normal goat serum with 0.2% Triton X-100. Primary antibodies: anti- glial fibrillary acidic protein (GFAP; 1:1000, Dako Z033401-2), anti-ionized calcium-binding adapter molecule 1 (Iba1; 1:1000, Novachem 019-9741), and anti-cleaved caspase-3 (CC3; 1:500, Cell Signalling 966L1). Secondary labelling used either donkey anti-rabbit Alexa Fluor 488 (1:500, Invitrogen, Cat # A32790), or biotinylated anti-rabbit, goat polyclonal (Vector Laboratories, Cat# VEBA1000), followed by biotin-streptavidin avidin–biotin complex (ABC) with 3,3′-diaminobenzidine (DAB) visualisation. Slides were counterstained with 4′,6-diamidino-2-phenylindole (DAPI) or mounted in distyrene-plasticizer-xylene (DPX) as appropriate.

### Image acquisition and quantification

All analyses were performed blinded to group. Slides were imaged using an Olympus VS120 scanner (10× for H&E; 20× for IHC/IF). For each marker, three sections per brain were analysed at the lesion maximum and ±⅓ lesion span from either side. Regions of interest (ROI) included the infarct core, border, and corresponding sham cortex (Supplementary Fig. 2). As previously reported,^40,44,47^ positive area (GFAP, Iba1) was quantified via pixel thresholding after background subtraction; CC3-positive cells were manually counted in matched ROIs with full details in Supplementary Methods.

### Statistical analyses

Data are presented as mean ± standard error of the mean (SEM). Analyses were performed in GraphPad Prism (v8, USA). A *P* < 0.05 was considered significant. One-way and two-way ANOVAs with Sidak’s post hoc tests were applied to behavioural and histological data, as described in the figure legends. Lesion metrics and atrophy were tested by two-way ANOVA; correlations between lesion volume and behaviour were assessed by Pearson’s *r*. Sex effects were analysed using two-way ANOVA with Sidak’s correction. Power analyses were conducted using G*Power (v3.1).^48^ Full statistical outputs, inclusion/exclusion criteria, and sample size rationale are reported in Supplementary Methods and Supplementary Tables.

## Results

### Severe focal cortical injury did not affect body weight or cause mortality

Across all litters (*n* = 4 per timepoint), body weight increased steadily from P10 to P22 in both sham- and Rose Bengal–treated pups (Table 1; Supplementary Fig. 3). Within each litter, pups were allocated to each group to control for within-litter effects. Litter size varied only from 12 to 14 pups, and there was no significant association between litter size and average litter body weight (r_s_ = 0.774, *P* = 0.50) at P10. Sham animals gained weight from 19.6 ± 0.6 g at P10 to 53.3 ± 2.3 g at P22, and comparable growth trajectories were observed in the 25, 40, and 60 mg/kg dose groups. Only 1 of 54 pups in the dose response study died: a sham rat, 24 h after surgery.

**Table 1:** Comparison of weight gain between sham and focal injury rat pups at postnatal days

	P10	P11	P17	P22
**Sham pup no. (*n*, *litters*)**	18 (*4*)	18 (*4*)	13 (*4*)	9 (*4*)
**Average sham body weight (g)**	19.6 ± 0.6	21.2 ± 0.6	33.3 ± 1.2	53.3 ± 2.3
**25 mg/kg injury group average body weight in grams (*pup n, litter n*)**	20.5 ± 0.7 (*n* = *12, 4*)	22.2 ± 0.7 (*n* = *12, 4*)	35.1 ± 1.7 (*n* = *8, 4*)	56.3 ± 3.8 (*n* = *4, 4*)
**Post-hoc *P*-value 25 mg/kg RB versus sham**	0.878	0.790	0.578	0.366
**40 mg/kg injury group average body weight in grams (*pup n, litter n*)**	18.6 ± 0.9 (*n* = *12, 4*)	20.3 ± 1.1 (*n* = *12, 4*)	31.9 ± 1.8 (*n* = *8, 4*)	51.7 ± 2.7 (*n* = *4, 4*)
**Post-hoc *P*-value 40 mg/kg RB versus sham**	0.782	0.872	0.749	0.841
**60 mg/kg injury group average body weight in grams (*pup n, litter n*)**	20.2 ± 0.7 (*n* = *12, 4*)	20.6 ± 0.8 (*n* = *12, 4*)	32.0 ± 1.6 (*n* = *8, 4*)	51.0 ± 1.5 (*n* = *4, 4*)
**Post-hoc *P*-value 60 mg/kg RB versus sham**	0.973	0.965	0.789	0.602

g, gram; *n*, number; P, postnatal day; RB, Rose Bengal; vs, versus.

Body weights (mean ± SEM) and *P*-values are from a two-way ANOVA, followed by Sidak's-adjusted multiple comparison test.

### The severity of the injury was proportional to the Rose Bengal dose

#### Infarct volume is maximal at 60 mg/kg

**Figure 2:**
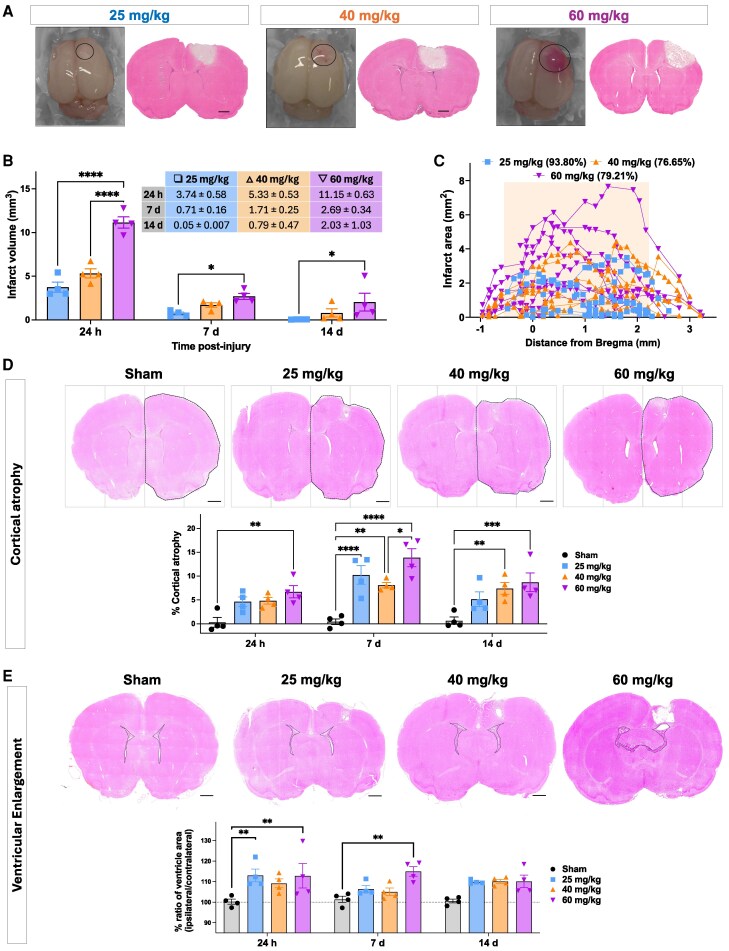
**Dose-dependent relationship of Rose Bengal with infarct volume, cortical architecture, and secondary injury.** (**A**) Macroscopic images of Rose Bengal-induced lesions (left) and representative haematoxylin and eosin (H&E)-stained coronal sections (right) showing dose-dependent infarct size and depth across 25, 40, and 60 mg/kg groups. Scale bar = 1 mm. (**B**) Quantification of infarct volumes at 24 h (h), 7 days (d), and 14 days across Rose Bengal doses. Data as mean ± SEM; *n* = 4 per timepoint per group, each rat is shown as individual points on the graphs; sex of each animal for each group outlined in Supplementary Table 11. Two-way ANOVA with Sidak's post-hoc test was used for multiple comparisons. *P < 0.05, ****P < 0.0001. Inset = lesion volume in millimetres cubed; ± SEM. (**C**) Infarct area plotted along the anterior-posterior axis relative to Bregma. Orange shading denotes the region of the primary motor cortex (+2.2 mm to −0.5 mm from Bregma). Each line represents one animal, and each point is a sectioned level through the brain (*n* = 12 per dose from four litters; see Supplementary Table 1). (**D, E**) Representative H&E-stained sections from sham, 25, 40, and 60 mg/kg groups, highlighting (**D**) cortical atrophy and (**E**) ventricular enlargement and quantification of (**D**) cortical atrophy and (**E**) ipsilateral ventricular volume across time points and doses (mean ± SEM; *n* = 4 pups per group from four litters, shown as individual points on the graphs; scale bar = 1 mm.). Two-way ANOVA with Sidak's post-hoc test was used for multiple comparisons. *P < 0.05, **P < 0.01, ***P < 0.001, ****P < 0.0001.

A two-way ANOVA revealed significant effects of Rose Bengal dose and time after injury across pups (*n* = 36; 4 pups/group per timepoint; Fig. 2A and B; Table 1, Supplementary Table 1). The infarct volume was largest at 24 h and smallest at 14 days post-injury for all Rose Bengal doses, with higher Rose Bengal doses producing larger infarcts (Fig. 2A and B).

#### The infarct is highly reproducible and consistently localized to the motor cortex in the 25 mg/kg Rose Bengal group

In the 25 mg/kg injury group, 93.8% of the infarct was in the primary motor cortex (Fig. 2C). At 40 and 60 mg/kg, the percentage within the primary motor cortex was 76.6% and 79.2%, respectively, relative to Bregma. Lesions were induced in all animals treated with Rose Bengal. Lesion reproducibility between litters was assessed at 24 h after injury, when we had the most data, by calculating the CV for the lesion volume (CV_lesion_) and the lesion area as a percentage of the hemisphere (CV_percent_; Supplementary Table 2). The CV was the lowest (i.e. the least variation between litters) in the 60 mg/kg group (CV_lesion_, 11%—CV_percent_ 16%), although, as outlined above, 30% of the lesion extended beyond the motor cortex (Fig. 2C). CV values across all groups ranged from 11% to 31%, within the previously defined high reproducibility range^49^ (Supplementary Table 2). The CV is also shown for the 7-day and 14-day recovery groups in Supplementary Table 2.

#### Infarct area correlates positively with infarct depth

A Spearman correlation coefficient test revealed a significant association between infarct area and depth at all Rose Bengal concentrations (Supplementary Fig. 4): 25 mg/kg Rose Bengal dose (r_s_ = 0.729; *P* < 0.0001), 40 mg/kg (r_s_ = 0.704; *P* < 0.0001) and 60 mg/kg (r_s_ = 0.669; *P* < 0.0001). Although Rose Bengal injury was primary cortical, subcortical white matter damage was observed in the 25, 40 and 60 mg/kg groups 24 h after injury. In the 25 mg/kg group, approximately 5% of the injury was outside the motor cortex, driven by injury in one rat pup. In the 40 and 60 mg/kg groups, more than 15% of injury was observed outside the motor cortex in the subcortical white matter, occurring in three pups in each group. Also, lesion territory spilled over into the somatosensory cortex for one pup in the 40 mg/kg group and for two pups in the 60 mg/kg group.

#### Cortical atrophy and ventricular dilation were dose-dependent

There was a significant effect of Rose Bengal dose and time after injury on cortical atrophy in the two-way ANOVA (Fig. 2D, Supplementary Table 3). Cortical atrophy significantly increased at day 7 and 14 post-injury compared to sham values (Fig. 2D). There was also a significant effect of Rose Bengal dose on ventricular volume in the two-way ANOVA (Fig. 2E, Supplementary Table 3). Specifically, the total volume of the ipsilateral ventricles was significantly larger at 24 h and 7 days post-stroke compared to sham-operated controls in the multiple comparison testing (Fig. 2E).

### Severe focal injury in the neonate caused acute cell death and gliosis

**Figure 3:**
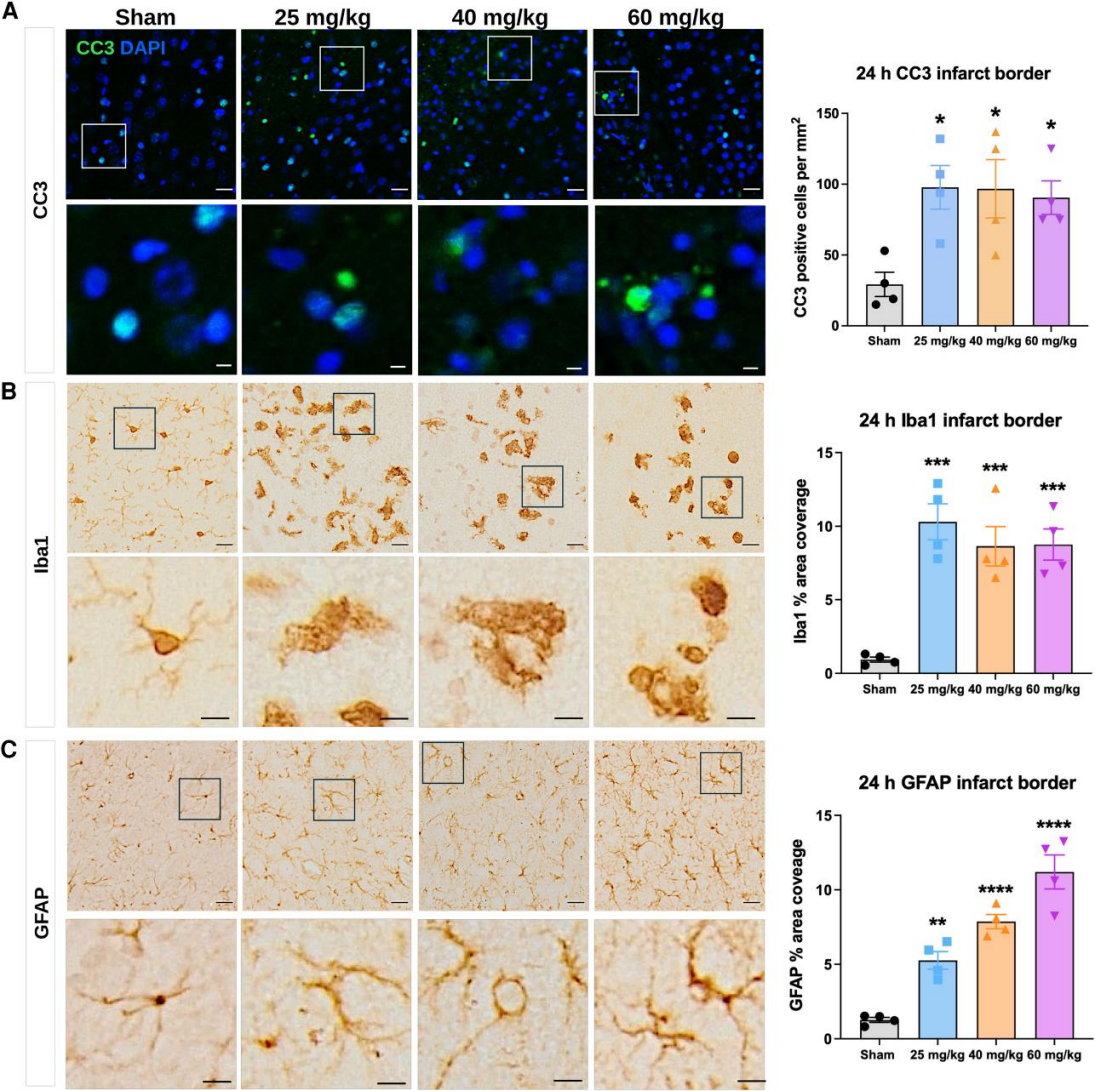
**Cell death and glial reactivity in the infarct border zone.** Representative images of (**A**) CC3, (**B**) Iba1 and (**C**) GFAP immunostaining in the infarct border at 24 h (h) post-injury and sham surgery, paired with quantification of total cell number per mm^2^ or the percent of glial reactive area covered. Data are presented as mean ± SEM; *n* = 4 litters; *n* = 4 pups per group, shown as individual points on the graphs; sex of each animal for each group outlined in Supplementary Table 11). One-way ANOVA with Sidak's *post-hoc* test was used for multiple comparisons. Scale bars, upper = 20 µm, lower = 5 µm; *P < 0.05, **P < 0.01, ***P < 0.001, ****P < 0.0001.

At 24 h post-injury, the number of CC3-positive cells and area coverage of Iba1 and GFAP were quantified within the infarct border area. We observed a significant increase in CC3 cell density (Fig. 3A), Iba1 area coverage (Fig. 3B), and GFAP area coverage (Fig. 3C) at the infarct border in all injury groups compared with sham; statistical outputs are in Supplementary Tables 4 and 6. There were no significant differences between the 25, 40, and 60 mg/kg groups in the width of the glial reactive zone for Iba1-positive cells (one-way ANOVA, Supplementary Fig. 5).

### Cell death and gliosis persisted after severe focal injury in the neonate

**Figure 4:**
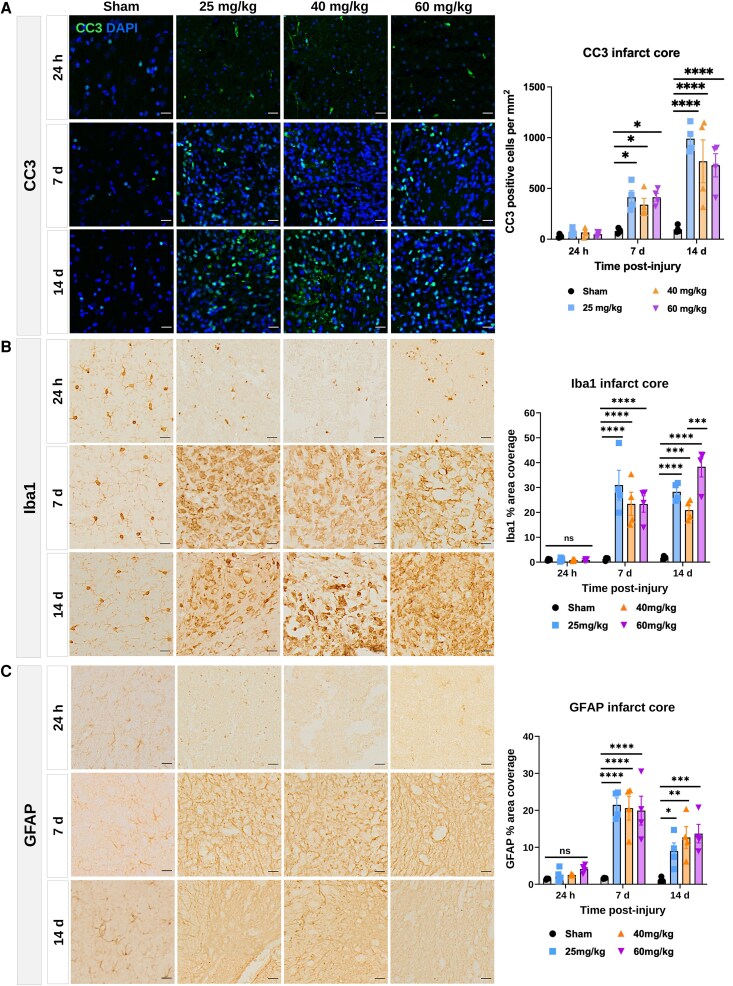
**Cell death and glial reactivity in the infarct core.** Representative images of (**A**) CC3, (**B**) Iba1, (**C**) GFAP in the core at 24 h (h), 7 days (d) and 14 days after injury, together with quantification of the cell density of CC3 and percentage of infarct core area covered by Iba1 and GFAP. Data presented as mean ± SEM. *n* = 4 litters: *n* = 4 pups per group, shown as individual points on the graphs; sex of each animal for each group outlined in Supplementary Table 11. Analysis using two-way ANOVA followed by Sidak's *post-hoc* test for multiple comparisons. *P < 0.05, **P < 0.01, ***P < 0.001, ****P < 0.0001, ns = not significant. Scale bars = 20 µm.

CC3, Iba1 and GFAP immunoreactivity were significantly increased in the infarct core at 7- and 14-days post-injury across all injury doses compared to sham controls based on a two-way ANOVA (Fig. 4A–C; Supplementary Tables 4, 6). There were no changes in CC3, Iba1 and GFAP immunoreactivity at 24 h after injury. Microglia/macrophages and astrocytes were recruited into the infarct core at later time points (7- and 14-day post-injury) following early localisation to the glial reactive zone at the border of the lesion.

### Severe focal cortical injury in neonatal rats impaired wire hang time and contralateral forepaw use in the days and weeks after injury

#### Wire-hang test

**Figure 5:**
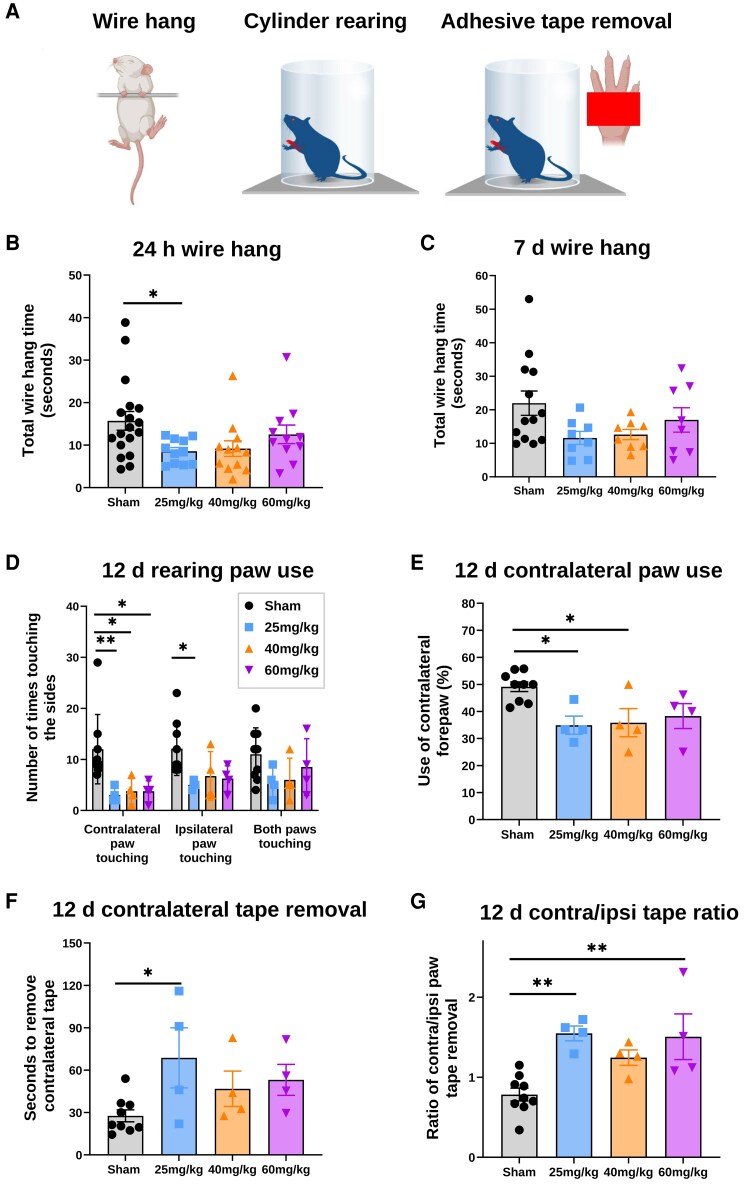
**Severe focal injury led to persisting behavioural deficits.** Changes were assessed using the (**A**) wire-hanging test, the cylinder rearing test, and the adhesive tape removal test. Wire hang data are shown at (**B**) 24 h (h) post-injury for sham (*n* = 18), 25 mg/kg (*n* = 11), 40 mg/kg (*n* = 12), and 60 mg/kg (*n* = 11) and (**C**) 7 days (d) post-injury for sham (*n* = 13), 25 mg/kg (*n* = 8), 40 mg/kg (*n* = 8), and 60 mg/kg (*n* = 8). Cylinder rearing data include (**D**) use of contralateral, ipsilateral, and both forepaws and (**E**) percentage of contralateral forepaw use at 12 days after injury at postnatal day (P)22 for sham (*n* = 9), 25 mg/kg (*n* = 4), 40 mg/kg (*n* = 4), and 60 mg/kg (*n* = 4). Adhesive tape removal data show (**F**) time to remove tape from the contralateral forepaw and (**G**) the ratio of contralateral/ipsilateral forepaw use at P22 for sham (*n* = 9), 25 mg/kg (*n* = 4), 40 mg/kg (*n* = 4), and 60 mg/kg (*n* = 4). Data are presented as mean ± SEM, with individual points shown for each rat analysed (minimum 4 litters). One-way and two-way ANOVA with Sidak's post-hoc test were used for multiple comparisons *P < 0.05, **P < 0.01. Created in BioRender. Fleiss, B. (2026) https://BioRender.com/wrmt0gc.

Injury significantly affected total hang time at 24 h and 7 days (one-way ANOVA). Sham pups hung longer than the 25 mg/kg group at 24 h (15.7 ± 2.2 s versus 8.6 ± 0.9 s; *P* = 0.039). There was also a trend toward reduced hang time at 24 h for the 40 mg/kg group (*P* = 0.058) and at 7 days for the 25 mg/kg group (*P* = 0.069; Fig. 5B and C; Supplementary Table 7).

#### Cylinder rearing

Injury altered forelimb use (one- and two-way ANOVA). Pups that received 25 mg/kg (but not 40 or 60 mg/kg) of Rose Bengal showed reduced use of the contralateral and ipsilateral forepaw 12 days after injury. Those given 25 or 40 mg/kg displayed contralateral forepaw hypokinesia, with significantly lower contralateral use than sham (Fig. 5D and E; Supplementary Table 7).

#### Adhesive-tape removal

Sensorimotor performance was impaired 12 days after injury (one-way ANOVA). Sham pups removed the tape faster (27.6 ± 4.2 s) than the 25 mg/kg group (68.7 ± 21.3 s; *P* < 0.05). No significant differences were found for the 40 mg/kg (46.8 ± 12.5 s) or 60 mg/kg (53.1 ± 11.0 s) groups (Fig. 5F). The contralateral/ipsilateral tape removal ratio was increased in the 25 mg/kg (1.5 ± 0.1) and 60 mg/kg (1.5 ± 0.3) groups compared with sham (0.8 ± 0.1; *P* < 0.01), but not at 40 mg/kg (1.2 ± 0.1; Fig. 5G; Supplementary Table 7).

No significant injury effects were observed in *open-field behaviour* (locomotion or time spent in the centre; Supplementary Fig. 6).

### Exploratory correlation between lesion volume and motor function

We next explored whether lesion volume correlated with motor performance to identify the optimal Rose Bengal dose. Among the eight behavioural measures in Fig. 5, six were significantly altered in the 25 mg/kg group but only two in the higher-dose groups, prompting a Pearson correlation analysis (Supplementary Table 8).

At 24 h, wire-hang performance showed there was no interaction with lesion volume in the 25 mg/kg (r = −0.53, *P* = 0.24) and 40 mg/kg groups (r = −0.72, *P* = 0.25), and no correlation at 60 mg/kg (r = −0.03, *P* = 0.49). Pooling the 25 and 40 mg/kg data increased power, revealing a strong trend between greater lesion volume and shorter hang time (r = −0.63, *P* = 0.06).

When wire-hang scores at 24 h were compared with lesion volume at 7 days, negative correlations emerged at 25 mg/kg (*r* = −0.81, *P* = 0.09) and 40 mg/kg (*r* = −0.99, *P* = 0.002), suggesting early motor deficits predict later tissue loss. For long-term outcomes, adhesive-tape removal performance at 12 days correlated strongly and significantly with lesion volume at 14 days in the 25 mg/kg group (*r* = −0.95, *P* = 0.03), whereas only weak, non-significant relationships were found at 40 mg/kg (*r* = −0.17, *P* = 0.42) and 60 mg/kg (r = −0.16, *P* = 0.42).

Together, these findings indicate that moderate-dose photothrombosis (25–40 mg/kg) yields behaviour–lesion relationships consistent with scalable, reproducible injury suitable for functional outcome studies.

### The impacts of severe focal brain injury were not sex specific

**Figure 6:**
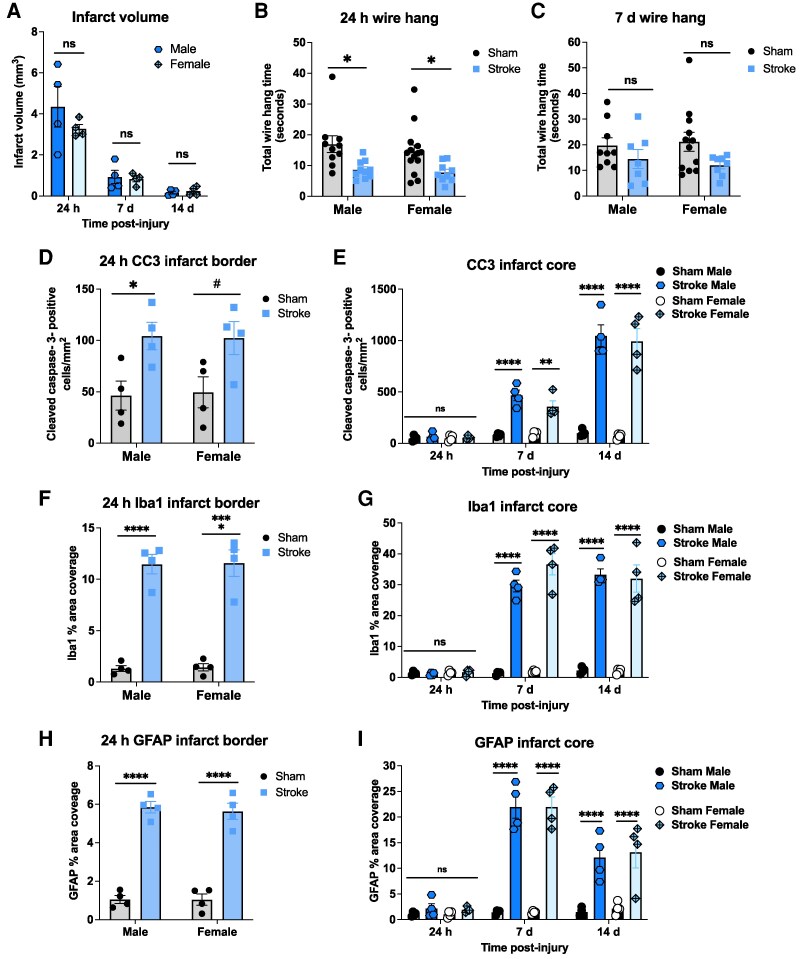
**No sex differences in the effects of injury on behaviour and neuropathology.** (**A**) Infarct volume quantified from H&E-stained brain sections at 24 h (h), 7 days (d), and 14 days post-injury (*n* = 4 per group). (**B**) Wire-hanging times at 24 h post-injury for sham and injured males (*n* = 10), sham females (*n* = 14), and injured females (*n* = 9), and (**C**) at 7 days post-injury in for sham males (*n* = 9), sham females (*n* = 12), injured males (*n* = 7), and injured females (*n* = 8). (**D**) CC3 positive cells in the infarct border at 24 h and (**E**) in the infarct core at 24 h, 7 days and 14 days (*n* = 4 per group). Area coverage in the infarct border at 24 h and in the infarct core at 24 h, 7 days and 14 days for (**F, G**) Iba1 and (**H, I**) GFAP (all *n* = 4). Analyses were performed using two-way ANOVA with Sidak's *post-hoc* test for multiple comparisons. Graphs show mean ± SEM; individual points for each animal analysed. *P < 0.05, **P < 0.01, ****P < 0.0001, ^#^*P* = 0.05–0.099; ns = not significant.

To address whether sex influenced lesion characteristics or behaviour, additional animals were added to the 25 mg/kg group to give *n* of 7–14 (Fig. 6). No significant differences were found between males and females in infarct volume (Fig. 6A), wire hang at 24 h or 7 days after injury (Fig. 6B), anxiety-related or locomotor behaviours (Supplementary Fig. 7), CC3 positive cells (Fig. 6C) or area coverage of Iba1 (Fig. 6D) or GFAP (Fig. 6E). Two-way ANOVA results are summarized in Supplementary Table 9. Power calculations were undertaken (G*Power, 3.1), Supplementary Table 10, to determine Cohen's effect size and the number of rats required to detect statistically significant sex differences in a two-tailed *t*-test (α = 0.05, power = 0.8). For infarct volume, the number of pups required at 24 h would be 29 pups, at 7 days would be 385, and at 14 days would be 80. For a wire hang at 24 h to detect a difference between the shams would require 297 pups, and between stroke would require 232 pups; at 7 days, it would require 801 to detect a difference between the sham and 174 between stroke pups. Accordingly, it would not be practical or ethical to conduct additional studies to examine sex-dependent effects on these outcome markers.

## Discussion

This study describes a minimally invasive, highly reproducible, scalable and specific form of motor cortex infarction in the neonatal brain that correlates with long-term functional impairment. A 19-minute surgery using a 25, 40 or 60 mg/kg dose of Rose Bengal activated by a 1 mm diameter light applied to the intact skin for 10 min resulted in a cortical lesion. The 25 mg/kg dose of Rose Bengal most reliably induced a lesion localized to the motor cortex and functional impairments, which correlated with lesion volume, a key factor in developing an early-phase screening model with translational potential.

Body weight did not differ between the sham and injury groups, likely due to the short procedure duration, the use of short-acting anaesthesia, and the modest lesion volume compared to other models. In the present study, lesion volumes at 24 h post-injury across Rose Bengal doses ranged from 3.7 to 11.1 mm^3^. Knezic and colleagues reported larger infarct volumes, 8.2 to 14.0 mm^3^ that were associated with weight loss in young adult mice following photothrombotic stroke.^28^ Similarly, infarct size strongly correlated with body weight loss in an adult mouse MCAO model.^50^ The lack of systemic effects on weight is advantageous in a high-throughput screening model, since it avoids confounding from weight loss or general poor health. Weight loss is also common in MCAO models, where anaesthesia typically lasts ∼90 min^51^ or repeated exposure is required.^52^ Anaesthesia itself can cause weight loss, even without other interventions,^53^ and it can also lead to brain cell death.^34^ Neonates may tolerate moderate cerebral infarction without affecting their weight, since maternal care mitigates stress and provides better nutrition than in adults, who must obtain food and water independently.^54^

An additional benefit of this model is that it avoids skin incision, thereby reducing overall harm to the animals, including post-operative burden, such as local inflammation and stress confounders. This contrasts with invasive neonatal photothrombotic models, which require jugular vein injection, a midline scalp incision, and craniotomy^31^ or, at a minimum, a skin incision to place the light source against the skull.^32^ In our protocol, light placement through the skin is possible because clear anatomical landmarks on the skull are visible, allowing reliable identification of Bregma. This minimally invasive approach also facilitates testing of therapies that themselves require surgical delivery at a different time or day, such as hydrogel implants.^4,33^ Furthermore, there was no mortality in the infarct group, compared to a rate of 2.45% in a model requiring a skin lesion for light placement^32^ and substantially lower than in models of MCAO, which can reach 40%.^24^ Finally, this procedure is amenable to high-throughput screening; up to 20 pups can be processed per morning by two researchers, allowing for multiple therapeutic agents to be screened relatively quickly.

To assess biological reproducibility, we calculated the coefficient of variation (CV) for each litter, treating it as a biological replicate. This approach avoids pseudoreplication and captures variability across independent applications of the model, which is important given that up to 60% of the variance in brain injury models arises from between-litter effects.^55^ CVs are rarely reported in perinatal injury studies, but for the Rice–Vannucci model, a CV of 54% has been noted.^56^ In adult stroke models, a review reported CV ranges of 50–150% for intraluminal MCAO, 80–100% for embolic stroke, and 5–30% for photothrombotic models.^49^ The CV is a practical index of model utility in preclinical screening: to detect a 30% difference between groups (80% power, α = 0.05), 16 animals per group are required at a CV of 30%, but 44 are required at a CV of 50%. Importantly, smaller lesion volumes inflate CV, as even minor deviations significantly affect the ratio; conversely, larger lesions reduce CV because proportional variability is lower. Accordingly, the 60 mg/kg dose was expected to produce a lower CV, but even at 25 mg/kg, the CV was within the highly reproducible range.

The photothrombotic lesion model produced hemispheric asymmetry and ventricular enlargement, reflecting tissue loss and chronic atrophy, consistent with outcomes observed in other neonatal injury models.^31,57-59^ Higher Rose Bengal doses led to greater ipsilateral hemisphere volume loss and enlargement of the lateral ventricle. Importantly, in babies and children following neonatal stroke, similar patterns of ventricular enlargement^57^ and hemisphere asymmetry^60^ are reported. As a result, this model is suitable for investigating potential neuroprotective or reparative therapies that aim to prevent chronic structural changes after severe ischaemic insults, such as neonatal strokes.

Consistent with ischaemic insult causing persistent injury, we found that a marker of apoptotic cell death, CC3, was increased at 24 h and remained elevated up to 14 days after injury. Elevated levels of cell death have been seen for at least 12 weeks after stroke in an adult rat model^61^ and in adult models of traumatic brain injury.^62^ However, although CC3 is widely regarded as a key apoptotic executioner, emerging evidence highlights its non-apoptotic roles, including involvement in reactive astrogliosis and macrophage infiltration.^63^ Increases in CC3 immunoreactivity may relate to ongoing neuronal or microglial apoptosis^64^ or as a specific inducer of a classically pro-inflammatory immune response in microglia in the region.^65^ We note that there was no apparent cell death in the core at 24 h, as complete blockage of the blood supply in this model leads to rapid necrotic cell death.^66^ In contrast, we speculate that at the later time points, resident cells that may have survived the initial injury, or glial cells that have migrated into the core as part of the secondary injury response, undergo delayed apoptotic processes.

Effective therapies for severe focal brain injury in the neonate must modulate the neuroinflammatory response, given its central role in injury progression,^67-69^ including the morphological transition and increased expression of Iba1 by microglia and macrophages and GFAP-positive glial scar formation.^29,32,70-74^ In our model, GFAP peaked at 7 days after injury and declined by 14 days, but remained significantly elevated compared to control levels. These findings are consistent with observations in models of severe neonatal focal brain injury, including HIE, neonatal stroke, or paediatric TBI in mice,^70,75^ rats,^32,76^ non-human primates,^77^ piglets,^78^ and sheep.^73^ Evidence suggests that the post-injury gliotic scar is less inhibitory to innate regeneration in the neonate. For instance, the post-stroke infant primate neocortex forms a smaller, more discrete chronic scar than in adults, correlating with greater neuronal sparing.^77^ Pro-regenerative neonatal astrocytes are also observed after injury to the neonatal spinal cord,^79^ setting the stage for further work to examine the phenotypic profile of these GFAP-reactive cells.

Ongoing microgliosis is another hallmark of perinatal brain damage and a potential target for the development of effective neurotherapeutics.^68,80^ In our model, macrophage/microglial density within the lesion core was dramatically elevated for at least 14 days, even as the lesion was reducing in volume. This could suggest a decoupling between tissue loss and inflammatory resolution, or that microglia are key in facilitating repair. Supporting the latter is the fact that depletion of microglia before injury in a rat MCAO model of neonatal stroke increased pro-inflammatory markers and lesion volume,^81^ but the role of microglia is complex.^82^ An alternative, and not mutually exclusive, explanation for the increase in macrophage/microglial density within the lesion core, together with the progressive reduction in lesion volume, is that a relatively stable, or only modestly changing, number of immune cells occupies a smaller tissue volume. Thus, the observed increase in density may reflect both continued localisation of macrophages/microglia to the most damaged tissue via chemotactic signalling and geometric effects arising from lesion consolidation, rather than solely ongoing cellular accumulation. These highlights that future studies should assess microglial phenotypic shifts in this model to determine whether they are predominantly deleterious or reparative, and whether function is context- and time-dependent, as well as markers of proliferation and programmed cell death in these cells.^68,83,84^

The present study found that higher doses of Rose Bengal increased the severity of brain damage, but this did not translate into a proportional increase in motor and sensory impairments, outcomes relevant to patients.^85^ This is unexpected, as damage to the white matter, including the corticostriatal projections, descending motor fibres, and sensorimotor integration pathways, has been shown to disrupt these behaviours.^32,86^ However, similar discrepancies between lesion volume and behavioural outcomes have been observed in young adult mice following photothrombotic stroke.^28,87^ These similar findings include that, in the tape-removal test following endothelin-induced injury, damage to the cortex alone resulted in deficits that persisted for 2 weeks, whereas damage to the cortex and striatum resulted in deficits for only 1 week.^88^ Variability in the behavioural phenotype has been observed in studies of the MCAO model, likely due to variability in the lesion territory,^89^ a limitation minimized in this model. One explanation for the injury-volume-behavioural disconnect may be that the behavioural tests used have limited sensitivity above a threshold of injury, due to lesion cross-over into other regions or changes in mood (motivation) and behaviour that affect how the animals undertake the behavioural paradigm.^90^ This may have masked subtle dose-dependent effects. We also speculate that white matter damage, when occurring in addition to cortical injury, may reach an injury threshold after which compensatory rerouting begins, so that additional functional impairment is not visible with the used tests.^91^ Future studies should incorporate quantitative sensitive assays, such as automated gait analysis or a balance beam, to expand our understanding of the relationship between lesion territory, size, and functional outcomes.

We found no significant sex differences in lesion volume, behavioural performance, or inflammatory responses in this model. This contrasts with clinical and most preclinical studies that report greater injury severity, higher mortality rates, and more severe sensory-motor impairments in neonatal males than females after ischaemic injury and in response to treatments,^44,92,93^ including in an adult model of photothrombotic infarct in the mouse.^94^ Our power calculations suggest that the effect sizes were large only for lesion volume at 24 h; even then, to achieve statistical significance, 29 rats per group would have been required, and the group sizes for the other analyses were inappropriate to consider. This suggests that, under our experimental conditions, sex effects are biologically minimal. Further studies with longer follow-up periods and detailed analyses (e.g. transcriptomic or circuit-level approaches) are required to fully assess the impact of sex on outcomes in this model.

Histological and behavioural assessments were conducted up to 14 days post-injury, which poses a potential limitation, as this period is equivalent to 2–3 years in humans.^95,96^ A longer follow-up would provide a more comprehensive understanding of both recovery and chronic pathology. Second, while the behavioural tests employed detected major sensorimotor impairments, more challenging tasks, such as the rotarod, staircase test, or complex wheel, may better capture subtle or higher-order functional deficits. Third, we did not include detailed molecular profiling of glial subtypes or phagocytic microglial/macrophage activation states, which could offer deeper insights into the inflammatory and reparative mechanisms at play.

Despite the limitations, the model provides key practical advantages: a simple, fast and minimally invasive procedure with excellent long-term survival, high reproducibility, scalable lesion size, and measurable neurofunctional deficits. The consistency of lesion size and territory also enables efficient parallel testing of candidate therapeutics while maintaining animal welfare, making it a valuable tool for studies of severe neonatal focal brain injury.

## Supplementary Material

fcag171_Supplementary_Data

## Data Availability

All data supporting the findings of this study, including raw and processed datasets, are available from the corresponding author upon reasonable request. Summary data and key analyses are provided in the main text and the supplementary materials. No proprietary software was used beyond standard packages.
