# Supplementary material for: A minimally invasive, scalable and reproducible neonatal rat model of severe focal brain injury
Source: Brain Commun. 2026 May 15;8(3):fcag171. doi: 10.1093/braincomms/fcag171 (PMC13201095; doi:10.1093/braincomms/fcag171)
Supplement: fcag171_Supplementary_Data [file fcag171_supplementary_data.pdf]

Materials and methods

Animals and surgical procedures

All experiments were approved by the RMIT University Animal Ethics Committee (AEC 1918), and work is reported in alignment with the ARRIVE guidelines. Rats were obtained from Australian Bioresources (Perth, Western Australia) at 12–16 days of pregnancy and allowed to deliver undisturbed. Body weights were recorded for all animals on postnatal (P) day 8, and each animal was assigned a random identifier. Then, using a restricted randomisation approach, they were group-allocated within litters in a block design. P10 was chosen for injury induction because it is considered approximately equivalent to a term-born human infant in terms of brain development.[1] Predetermined exclusion criteria included situations in which animals needed to be euthanised prior to planned experimental endpoints for humane reasons, or technical issues with tissue processing or staining. Only one animal died before the assigned collection point (sham group), and no other animal was excluded at any point across the analyses. For neuropathological assessments, one animal per litter per time point was included. For behaviours, each pup in the litter was tested and included as an independent replicate. For sex analysis, additional animals were added to the 25 mg/kg group.

P10 male and female Wistar rats were anaesthetised initially with 5% isoflurane (Isoflo, Abbott, NSW, Australia) in 0.4 L/minute oxygen by inhalation from a face mask for 2 minutes, then anaesthesia was maintained with 2–3% isoflurane in 0.4 L/minute oxygen. Body temperature was maintained using a thermistor-controlled heating pad (Thorlabs, USA). Rose Bengal (Merck, Australia, cat#330000), dissolved in sterile saline solution and protected from light was injected intraperitoneally at 25, 40 or 60 mg/kg, and after 5 minutes, a 1 mm diameter laser emitting diode (LED) light source (Thorlabs, US, cat# M565F3, 565 nm) mounted on the arm of the stereotactic unit was placed on the skin 2 mm to the right of Bregma and illuminated on full power for 10 minutes to induce a photothrombotic cortical injury. Doses of Rose Bengal were selected to span a range of possible injury severities, based on a review of the literature[2,3].

When viewed from above, Bregma appears as a dark patch at the intersection of an imaginary line drawn between the medial canthus (inner eye crease) and the posterior border of the ear lobe when viewed from above (**Supplementary Fig. 1**). At the end of the surgery, which took on average 19 minutes, buprenorphine (0.05 mg/kg; stock 300 µg/mL in sterile saline, Cenvet, Australia) and meloxicam (5 mg/kg; stock 5 mg/mL in sterile saline, Cenvet, Australia) were injected subcutaneously, and after a further 60 minutes of recovery on a pre-warmed heating pad, pups were returned to the dam. Sham controls were administered Rose Bengal without laser illumination to account for the confounding effects of the dye,

which can affect the vasculature.[4] Light exposure has no reported discernible effects and was not considered a confounding factor.

Behavioural testing

Behavioural testing was undertaken between 0800 and 1200 hours. Rats were identified only via the number allocation from P8. Rats were individually transferred from their home cage to a holding cage, then to the testing area. All behavioural equipment was thoroughly cleaned with 70% ethanol and allowed to dry to eliminate residual odours between test sessions. The testing scheme is summarised in **Fig. 1**. P11 (24 hours post-injury) was selected to capture the acute phase of neuroinflammation and glial activation, corresponding to a stage in rodent brain development broadly equivalent to a human term infant. P17 (7 days post-injury) reflects the subacute phase, during which lesion consolidation and active remodelling occur, aligning with the end of the neonatal period in humans. P24 (14 days post-injury) represents the early recovery phase, coinciding with a surge in myelination and cortical circuit maturation in rats, developmentally analogous to late infancy to early toddlerhood (approximately 2–3 years in humans).[1,5,6]

Wire hang test. Wire hang tests were conducted at P11 and P17. The rats were held by the scruff of the neck skin and supported until they grasped the wire with both forepaws. They were allowed to hang for a maximum of 5 minutes. Three separate hang times were recorded for each rat, with a 30-second rest period between each trial. Trials were recorded, and the total time spent hanging on the wire was logged from the video footage. The distance from the wire to the surface below was approximately two body lengths at P11 (i.e., 24 hours after inducing the injury), and approximately three body lengths at P17 (7 days after the injury).

Cylinder rearing test. At P22 (i.e., 12 days post-injury), rats were placed in a transparent plexiglass cylinder (185 mm height x 130 mm outer diameter). Video footage was recorded for 5 minutes using a camera positioned beneath the cylinder, capturing an upward view of the rat. The number of times the rat reared onto its hindlimbs and contacted the cylinder with one or both forepaws was recorded. Wall touches were categorised and counted separately for each forepaw (right and left) as well as for simultaneous bilateral contacts (double contact). Data were expressed as a percentage of the impaired forelimb touches relative to total forelimb contacts, calculated as: $= \left(\frac{(contralateral\ touches)}{(ipsilateral\ touches + contralateral\ touches)}\right) \times 100$.

Adhesive tape removal test. At P22, two identical pieces of adhesive tape (Tough-Spots, 0.95 cm diameter, halved, Merck, USA, Z743588) were affixed to the hairless area of the palm of each forepaw. Rats were

immediately placed in a transparent plexiglass cylinder (185 mm height x 130 mm outer diameter) with video recording from underneath. Each test session consisted of three trials, with a 1-minute rest period between each. Each trial had a maximum duration of 3 minutes; if the tape was not removed within this period, the trial was recorded for up to three minutes. Initial contact time was defined as the first instance of paw shaking or bringing the paw to the mouth. Sensorimotor performance was assessed by measuring the time taken to remove the adhesive from each paw.

Open field test. At P11 and P17, rats were placed in the centre of a square arena (30 x 30 cm) and allowed to explore freely for 3 minutes, recorded via an overhead camera. Parameters analysed included horizontal activity (number of line crossings), time spent in the centre and periphery zones, immobility duration, and rearing behaviour.

Brain tissue collection and processing

Brains were collected from injured and sham rats at P11, P17, and P24 (**Fig. 1**). Rats were anesthetised with an intraperitoneal injection of Lethabarb (325 mg/mL solution, >200 mg/kg, Virbac, Australia, plus 10 mg/mL Lignocaine, Cenvet, Australia) and transcardially perfused with 0.1 M phosphate buffered saline (PBS, pH 7.4) for 1 minute followed by 4% paraformaldehyde (PFA, Sharlau, Barcelona, Spain), for 4 minutes at a flow rate of approximately 2 mL/minute. Brains were post-fixed for 12-18 hours at 4°C in a 1:10 volume of PFA, then transferred to 70% ethanol. Tissues were processed (Leica Microsystems, Australia, model ASP300S) and embedded in paraffin wax in a coronal orientation. Processing conditions were 1 hour each in the following: 70% ethanol, 90% ethanol, 100% ethanol, 50/50 ethanol/xylene, xylene, fresh xylene, paraffin wax, fresh paraffin wax, and fresh paraffin wax. Coronal sections were cut on a rotary microtome (Thermoline Scientific, Australia, model HM325), starting at approximately Bregma +3.24, before the lesion appeared, and continuing through the brain until no lesion was present. Sections were cut at 8 µm, transferred to float in a 37°C water bath, and every twentieth section (three sections per slide, spaced 160 µm apart) was mounted onto Superfrost Plus slides (Menzel-Gläser, Germany) and air-dried before storage.

Infarct, cortical atrophy and ventricle asymmetry analysis

Haematoxylin and Eosin (H&E) staining. Every twentieth section throughout the lesion was stained. The slides were deparaffinized, rehydrated, stained in 0.3% Mayer's Haematoxylin (Amber Scientific,

Australia) for 4 minutes, washed in running tap water until the water was colourless, and then submerged in Scott's tap water (20 g of magnesium sulphate and 2 g of sodium bicarbonate, both from Sigma Aldrich, in 1 litre of deionized water) for 1 minute. Sections were then rinsed in running tap water again for 3 minutes, counterstained with 1% alcohol eosin (Amber Scientific, Australia) for 3 minutes, dehydrated and cover-slipped with dibutylphalate polystyrene xylene (DPX; Merck, Australia).

Infarct volume, location and depth analysis. Images were collected at x10 magnification using the Olympus VS120 Virtual Slide Microscope (Olympus Life Science, VIC, Australia). All analyses were performed by experimenters blinded to group assignments. The area and depth of the infarct were measured using ImageJ (ImageJ 1.53c Wayne Rasband, National Institutes of Health, USA) as previously.[7,8] The total infarct volumes were estimated using Cavalieri's principle by the following formula: $V = SA \times P \times T$, where V is the total volume, SA is the sum of areas measured, P is the inverse of the sampling fraction (20), and T is the section thickness (8 µm).

According to the Paxinos and Watson rat brain atlas [9], the area of the lesion from each section was plotted against its location relative to Bregma to assess the reproducibility of lesion location. The infarct area within the primary motor cortex was calculated using this formula: $= \left(\frac{Infarct\ area\ located\ in\ the\ primary\ motor\ cortex}{Total\ infarct\ area}\right) \times 100$. Infarct depth was measured from the mid-point of the lateral-to-medial edge of the lesion on the outermost cortical surface to the deepest visible lesion using the line tool in ImageJ.

The reproducibility of the lesion between litters of rats was assessed by calculating the coefficient of variation (CV) [10], as $\left(\frac{standard\ deviation}{mean}\right) \times 100$ for the lesion volume (CV_{lesion}), and the lesion area as a percentage of the hemisphere ($CV_{percent}$), expressed as a percentage.

Analysis of cortical atrophy and ventricle asymmetry. Brain atrophy in the affected right hemisphere was quantified by measuring the area of every 20th H&E-stained section and comparing it to the contralateral hemisphere. Cortical atrophy was calculated for each section using the formula: Cortical atrophy (%) = $\left[1 - \left(\frac{ipsilateral\ area}{controlateral\ area}\right)\right] \times 100$, representing the percentage of ipsilateral hemisphere atrophy, as previously [8,11]. Values were averaged across all analysed sections. Lateral ventricle size was also measured, and the lateral ventricle ratio (LVR) was calculated as the ipsilateral to contralateral lateral ventricle area ratio using ImageJ.

Immunohistochemistry (IHC) and immunofluorescence (IF)

Coronal sections spaced 160 μm through the lesion were dewaxed, rehydrated, and subjected to antigen retrieval as previously described.[12,13] All sections to be compared were processed within the same run to prevent inter-batch variation. Antigen retrieval was performed in citrate buffer (pH 6.0) heated to boiling in a microwave (Whirlpool M542), followed by two 7-minute heating cycles at 80% power with a 5-minute interval, and cooling in buffer for 20 minutes.

For IF, sections were washed in PBS (3 × 5 minutes), incubated in 0.1% Sudan Black B (Abcam, MA, USA; 0.1% in 70% ethanol) for 20 minutes to reduce autofluorescence, washed again, and blocked in 5% normal goat serum (NGS; ThermoFisher, Australia, cat# 10000C) with 0.2% Triton X-100 (Sigma-Aldrich, Australia, cat# 10789704001) in PBS for 1 hour at room temperature. For IHC, endogenous peroxidase activity was quenched with 3% H_2O_2 (Chem-Supply, Australia, cat# HA154-2.5L-P), then sections were blocked with 5% NGS and 0.2% Triton X-100 in PBS for 1 hour.

Sections were incubated overnight at 4 °C in a humidified chamber with the following primary antibodies diluted in 1% NGS/0.1% Triton X-100: anti-GFAP (rabbit polyclonal, 1:1000; Dako, cat# Z033401-2), anti-Iba1 (rabbit polyclonal, 1:1000; Novachem, cat# 019-9741), and anti-cleaved caspase-3 (rabbit polyclonal, 1:500; Cell Signalling, cat# 966L1).

The following day, IF sections were washed and incubated for 1 hour at room temperature with donkey anti-rabbit Alexa Fluor 488 secondary antibody (Invitrogen, cat# A32790), followed by DAPI nuclear counterstaining (ThermoFisher, USA; cat# D2149; 1:1000, 15 minutes). IHC sections were incubated for 1 hour with goat anti-rabbit biotinylated secondary antibody (Vector Laboratories, cat# VEBA1000), followed by Vectastain ABC Elite (Vector Laboratories, cat# PK6100; 1:1:200) and visualisation with DAB (50 mg in 10 mL dH_2O plus 3 μL 30% H_2O_2). All slides were washed thoroughly between steps (3 × 5 minutes).

IF sections were mounted in Vectashield aqueous medium (Vector Laboratories, USA) and protected from light. IHC slides were dehydrated and coverslipped with DPX. Slides dried for 24–48 hours before imaging.

Image analysis

Image acquisition and processing. Images were acquired using an Olympus VS120 slide scanner at x10 magnification for H&E and at x20 for IHC and IF. The exported data were analysed using ImageJ (ImageJ 1.53c Wayne Rasband, National Institutes of Health, USA), Excel (Microsoft 365, USA), and Prism (version 8, GraphPad Software Inc., USA).

Quantification of immunohistochemically stained areas. All analyses were performed blinded to group allocation. Pilot experiments at 6, 24, and 48 h and at 7 and 14 d post-injury were used to determine optimal time points and regions of interest (ROIs; **Supplementary Fig. 2**). For each marker, three coronal sections were analysed, at the lesion's maximal extent and one-third of either side.

At 24 h post-injury (P11), Iba1-positive microglia/macrophages were quantified in the infarct border (polygonal ROI), infarct core (polygonal ROI), and a fixed-size ROI in the corresponding sham cortex. At 7 and 14 days (P17, P24), Iba1 was assessed in the infarct core and sham cortex. GFAP-positive astrocytes and CC3-positive apoptotic cells were quantified at P11 in two fixed-size ROIs (200 µm × 400 µm) positioned laterally along the astrocyte reactivity zone, within the infarct core, and in the sham cortex; at later time points, these markers were analysed in the core and sham regions only.

The infarct border was defined as tissue surrounding the core between 24 and 48 h post-injury, whereas the core itself showed grossly altered morphology, acellular at early stages and densely glial at later stages. Astrocytic scar formation and microglial/macrophage accumulation were delineated using the polygon tool in ImageJ, as illustrated in **Supplementary Fig. 2**. The percentage area of Iba1- and GFAP-positive staining was determined by applying a consistent pixel-intensity threshold to 8-bit converted images. Background intensity was calculated from representative samples across groups, averaged, and then subtracted uniformly. The proportion of positive pixels was expressed as the number of thresholded pixels within the ROI relative to the total ROI area. CC3-positive cells were counted within the same ROIs used for GFAP analysis.

Statistical analyses

Data are presented as mean ± standard error of the mean (SEM). Statistical analyses were performed using Prism (version 8; GraphPad Software Inc., USA). A p-value < 0.05 was considered statistically significant. Parametric tests were used unless otherwise stated, based on their robustness to modest deviations from normality and the limited power of non-parametric alternatives in small *n* datasets. Infarct volume, cortical atrophy, and ventricular enlargement were analysed using two-way ANOVA followed by a Sidak's post-hoc multiple comparisons test. The relationship between infarct area and depth was assessed using Spearman's rank correlation coefficient (ρ), with corresponding r_s and p values reported. Differences in total wire hang time, cylinder rearing time, and adhesive tape removal time between the sham and injury groups were analysed using one-way or two-way ANOVA with Sidak's post-hoc multiple comparisons when a significant interaction or main effect was observed, as indicated in the figure legends. The number of

cylinder wall touches by forepaws was analysed using two-way ANOVA with Sidak's post-hoc test, where there was a significant interaction or main effect. CC3, Iba1 and GFAP staining in the infarct border were assessed using one-way ANOVA, while staining in the infarct core was analysed using two-way ANOVA with Sidak's multiple comparisons, as indicated in the figure legends. Correlations between behavioural outcomes and lesion volumes were assessed using Pearson's correlation coefficient. Sex-specific analyses were performed using two-way ANOVA with Sidak's post-hoc test. ANOVA main effects are reported in the **Supplementary Tables**, and the results of post-hoc tests are shown in the figures. Power analyses were performed using G*Power (version 3.1).[14]

References

1. Semple BD, Blomgren K, Gimlin K, Ferriero DM, Noble-Haeusslein LJ. Brain development in rodents and humans: Identifying benchmarks of maturation and vulnerability to injury across species. *Prog Neurobiol* 2013; **106-107**: 1-16.
2. Yi YY, Shin HJ, Choi SG, et al. Preventive Effects of Neuroprotective Agents in a Neonatal Rat of Photothrombotic Stroke Model. *Int J Mol Sci* 2020; **21**(10).
3. Jithoo A, Penny TR, Wen SW, et al. Effects of Light Exposure duration on Severity and Long-Term Neurodevelopment following Photothrombotic Stroke in a Neonate. *Dev Neurosci* 2025: 1-20.
4. Watson BD, Dietrich WD, Busto R, Wachtel MS, Ginsberg MD. Induction of reproducible brain infarction by photochemically initiated thrombosis. *Ann Neurol* 1985; **17**(5): 497-504.
5. Workman AD, Charvet CJ, Clancy B, Darlington RB, Finlay BL. Modeling transformations of neurodevelopmental sequences across mammalian species. *J Neurosci* 2013; **33**(17): 7368-83.
6. Clancy B, Kersh B, Hyde J, Darlington RB, Anand KJ, Finlay BL. Web-based method for translating neurodevelopment from laboratory species to humans. *Neuroinformatics* 2007; **5**(1): 79-94.
7. Chhor V, Moretti R, Le Charpentier T, et al. Role of microglia in a mouse model of paediatric traumatic brain injury. *Brain Behav Immun* 2017; **63**: 197-209.
8. Moretti R, Chhor V, Bettati D, et al. Contribution of mast cells to injury mechanisms in a mouse model of pediatric traumatic brain injury. *J Neurosci Res* 2016; **94**(12): 1546-60.
9. Paxinos G, Watson C. The rat brain in stereotaxic coordinates. Hard cover edition, vol. 1. London, UK: Elsevier Academic Press; 2013.
10. Yao H, Sugimori H, Fukuda K, et al. Photothrombotic middle cerebral artery occlusion and reperfusion laser system in spontaneously hypertensive rats. *Stroke* 2003; **34**(11): 2716-21.
11. Fleiss B, Nilsson MK, Blomgren K, Mallard C. Neuroprotection by the histone deacetylase inhibitor trichostatin A in a model of lipopolysaccharide-sensitised neonatal hypoxic-ischaemic brain injury. *J Neuroinflammation* 2012; **9**: 70.
12. Romantsik O, Ross-Munro E, Gronlund S, et al. Severe intraventricular hemorrhage causes long-lasting structural damage in a preterm rabbit pup model. *Pediatr Res* 2022; **92**(2): 403-14.
13. Bokobza C, Joshi P, Schang AL, et al. miR-146b Protects the Perinatal Brain against Microglia-Induced Hypomyelination. *Ann Neurol* 2022; **91**(1): 48-65.
14. Faul F, Erdfelder E, Buchner A, Lang AG. Statistical power analyses using G*Power 3.1: tests for correlation and regression analyses. *Behav Res Methods* 2009; **41**(4): 1149-60.

Supplementary figures

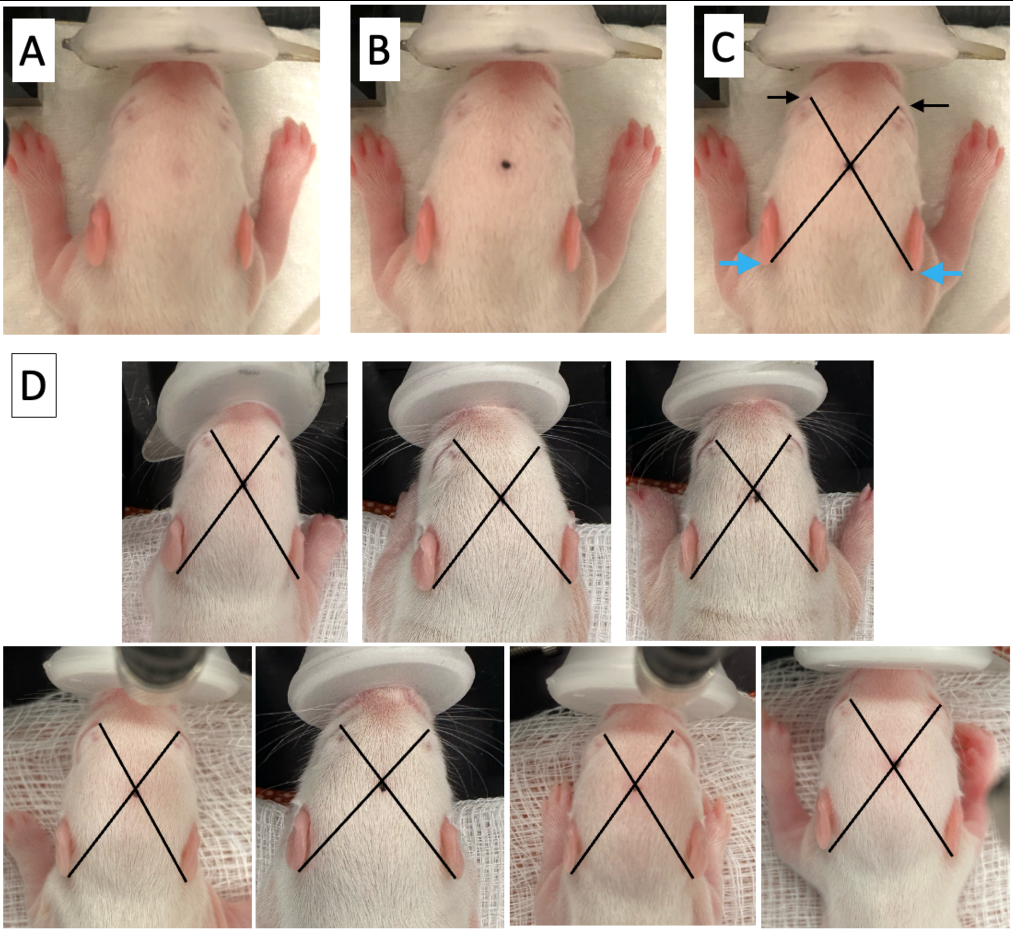

Supplementary Figure 1. Visualisation of the identification of Bregma in the P10 rats The view onto the top of the rat pups head, showing the (**A**) reliably present darker patch of hair that can be (**B**) used for marking our bregma which (**C**) lays on a diagonal line between the medial canthus (inner eye crease, black arrows) and the posterior border of the ear lobe (blue arrows) when viewed from above. (**D**) Examples of the location of the bregma relative to the anatomical regions from across experiments.

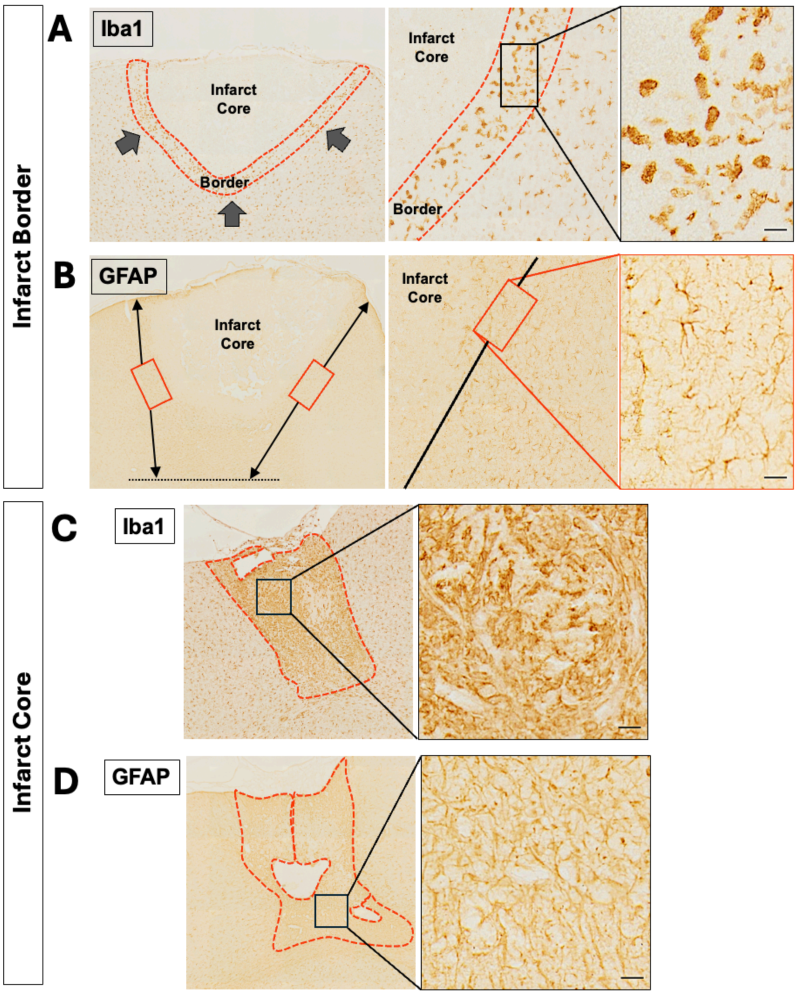

Supplementary Figure 2. Explanation of the locations of the zones of interest for Iba1 and GFAP staining analyses. In (**A**), the polygon tool was used to trace the band of ameboid Iba1-positive cells at the infarct border at 24 hours. Also shown is an inset of the infarct border zone with arrows indicating its width (upper left), followed by higher-magnification insets showing the inner border zone and the morphology of Iba1-positive cells (upper right). In (**B**), a representative image of GFAP staining at 24 hours, showing the placement of the two fixed-size regions of interest (ROIs) in the lesion by drawing straight lines from the upper cortical injury border to the lower injury border. The ROIs were placed halfway between the upper and lower infarct boundaries. A higher-magnification inset shows the GFAP infarct border zone and the morphology of GFAP-positive cells (lower right). In (**C**), the infarct core zone is present at all timepoints analysed in Iba1 (upper) and in (**D**) GFAP (lower) stained sections. The infarct core zone was delineated using the polygon tool on a low-magnification overview of the lesion, characterised by a densely microglia/macrophage- and astrocyte-populated area (left). Higher-magnification insets (right panel) show reactive glial cells within the infarct core. Scale bars = 20 µm.

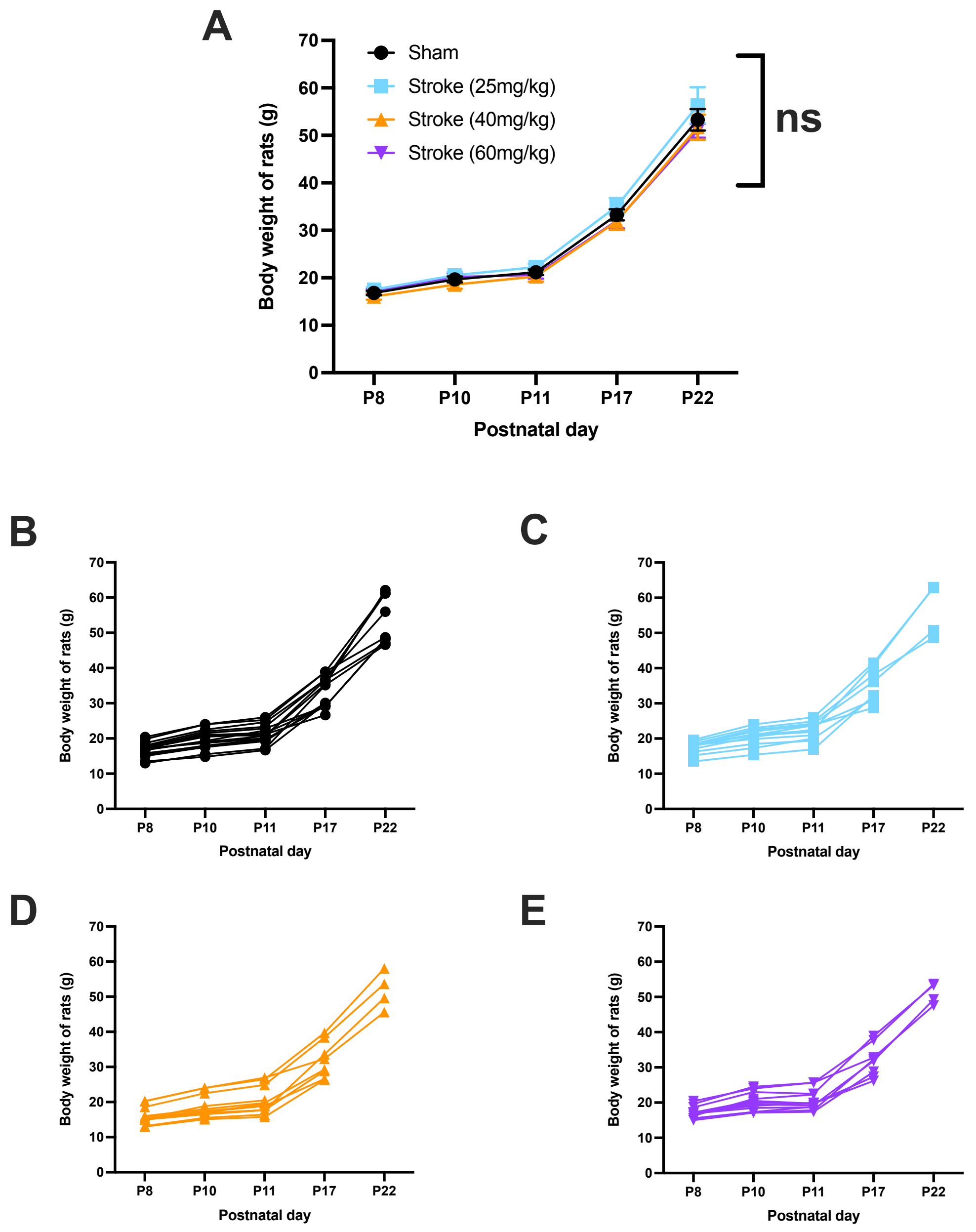

Supplementary Figure 3. Weight gain over time. In (**A**), weight is graphed for rats in the sham and the three Rose Bengal groups (mean ± SEM). Shams: P10, n = 18 from 4 litters; P11, n = 18 from 4 litters; P17, n = 13 from 4 litters; P22, n = 9 from 4 litters. 25 mg/kg groups: P10, n = 12 from 4 litters; P11, n = 12 from 4 litters; P17, n = 12 from 4 litters; P22, n = 8 from 4 litters. 40 mg/kg groups: P10, n = 12 from 4 litters; P11, n = 12 from 4 litters; P17, n = 12 from 4 litters; P22, n = 4 from 4 litters. 60 mg/kg groups: P10, n = 12 from 4 litters; P11, n = 12 from 4 litters; P17, n = 12 from 4 litters; P22, n = 8 from 4 litters. Statistical analysis was performed with a two-way ANOVA. ns = not significant. For the Main effects (F (Dfn, Dfd), p value: Interaction: (F (12, 200) = 0.419, p = 0.954; Rose Bengal dose: (F (3, 200) = 4.417, p = 0.004; Time post-stroke: (F (4, 200) = 480.3, p < 0.0001. In (**B-E**), weight gain shown for each individual rat to show variance.

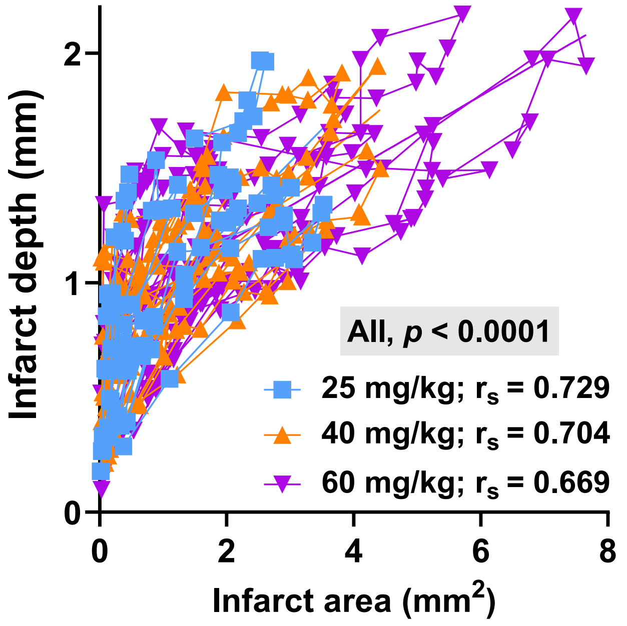

Supplementary Figure 4. Linear correlations between infarct area and infarct depth by dose across all timepoints. Spearman's correlation coefficient (r_s) and p-values shown. Each line is data from one animal; all groups are n = 4 per Rose Bengal dose per timepoint.

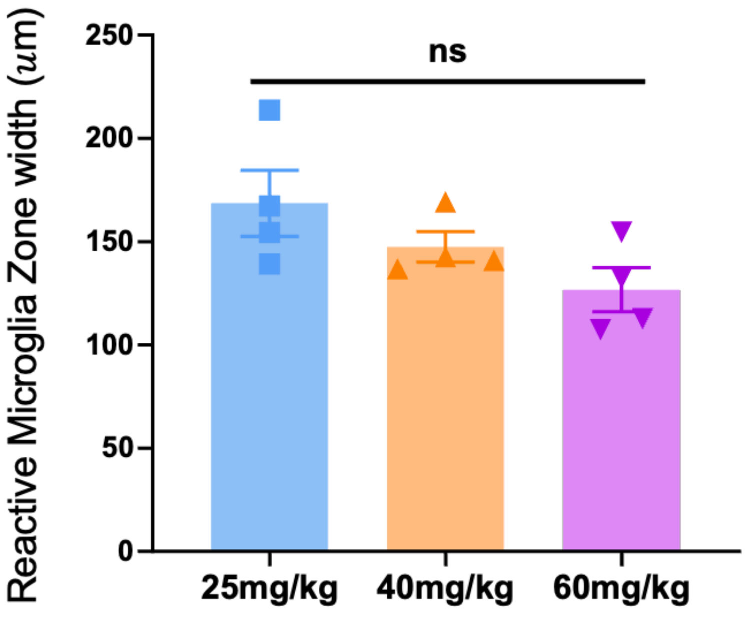

Supplementary Figure 5. Ameboid Iba1-IR zone width measurements at 24 hours post-injury. n = 4 independent replicates (rats from different litters) for each dose. Data are mean ± SEM, ns = not significant from one-way ANOVA, Sidak's *post-hoc* analysis. Results from the statistical tests are reported in Supplementary Table 5.

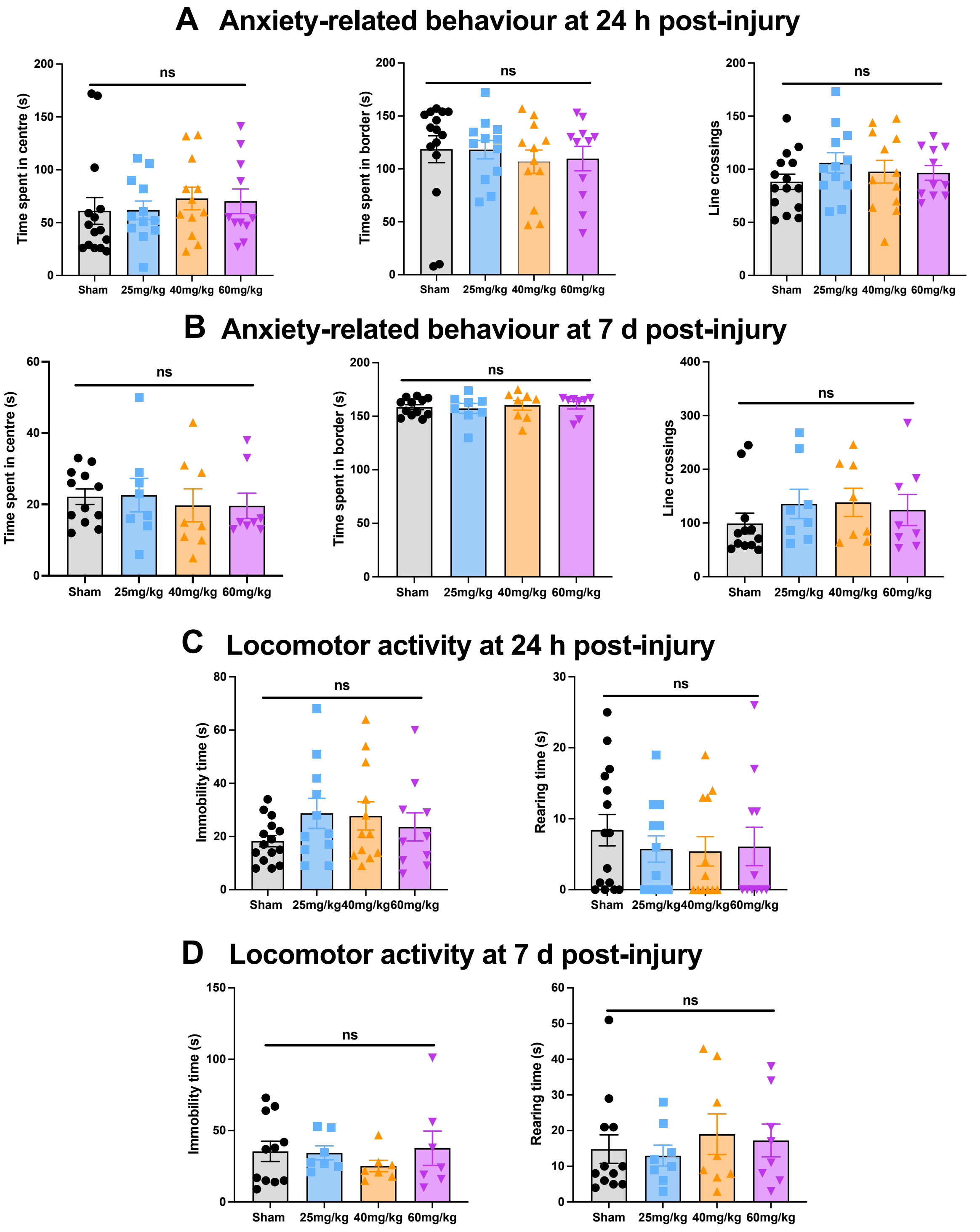

Supplementary Figure 6. Assessment of the behavioural changes in the open field test following neonatal injury. Data from 24 hours (**A**, **C**) and 7 days post-injury (**B**, **D**). Sham (n = 15, from 4 litters), 25 mg/kg (n = 12, from 4 litters), 40 mg/kg (n = 12, from 4 litters), 60 mg/kg (n = 11, from 4 litters) at 24h post-injury and Sham (n = 12, from 4 litters), 25 mg/kg (n = 8, from 4 litters), 40 mg/kg (n = 8, from 4 litters), 60 mg/kg (n = 8, from 4 litters) at 7d post-injury. Data are mean ± SEM, ns = not significant from one-way ANOVA, Sidak's *post-hoc* analysis. Results from the statistical tests are reported in Supplementary Table 7.

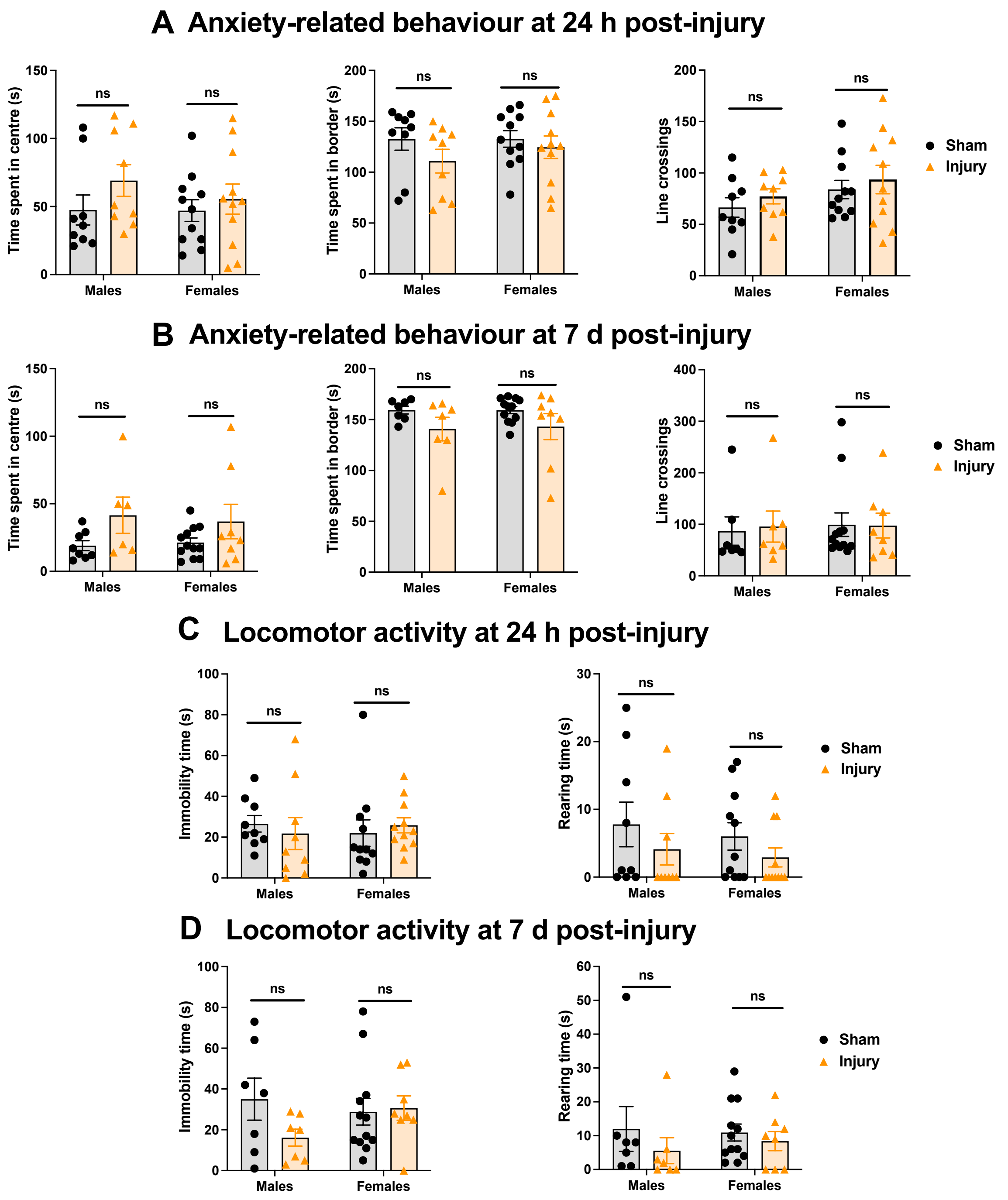

Supplementary Figure 7. No sex differences in the open field test following neonatal injury. Data from 24 hours (**A**, **C**) and 7 days (**B**, **D**) in males and females. At 24 hours post-injury from across four litters of rats, sham males (n = 9), sham females (n = 11), injury males (n = 9), injury females (n = 11), at 7 days, sham males (n=8), sham females (n = 12), injury males (n = 6), injury females (n = 8) post-injury. Data are mean ± SEM, ns = not significant from Two Way ANOVA, Sidak's *post-hoc* analysis. Results from the statistical tests are reported in Supplementary Table 9.

Supplementary tables

Supplementary Table 1. Results of the statistical analysis for infarct volume comparisons

Figure	Test name	Main effects			Sidak's multiple comparison			
		Interaction F (DFn, DFd), p value	Rose Bengal dose, F (DFn, DFd), p value	Time post-injury, F (DFn, DFd), p value	Time post-injury	Rose Bengal Dose comparisons	95% CI	p value
**Figure 2B,** Infarct volume	Two-way ANOVA	F (4, 27) = 10.43, ***p < 0.0001***	F (2, 27) = 40.78, ***p < 0.0001***	F (2, 27) = 106.1, ***p < 0.0001***	24 h	25 mg/kg vs 40 mg/kg	-3.492 to 0.3142	0.1233
						25 mg/kg vs 60 mg/kg	-9.321 to -5.515	***< 0.0001***
						40 mg/kg vs 60 mg/kg	-7.732 to -3.927	***< 0.0001***
					7 d	25 mg/kg vs 40 mg/kg	-2.898 to 0.9080	0.4772
						25 mg/kg vs 60 mg/kg	-3.881 to -0.07525	***0.0397***
						40 mg/kg vs. 60 mg/kg	-2.886 to 0.9197	0.4872
					14 d	25 mg/kg vs 40 mg/kg	-2.643 to 1.163	0.7011
						25 mg/kg vs 60 mg/kg	-3.880 to -0.07413	***0.0399***
						40 mg/kg vs 60 mg/kg	-3.140 to 0.6654	0.2938

Infarct volume data were analysed using two-way ANOVA to compare three Rose Bengal doses at multiple timepoints, with significance set at $p < 0.05$. Sidak's post-hoc test was used for multiple comparisons. **Bold and italicised values indicate statistically significant differences.** Sample sizes: 25 mg/kg = 4 rats, 40 mg/kg = 4 rats, 60 mg/kg = 4 rats. **Abbreviations:** ANOVA, analysis of variance; CI, confidence interval; DFd, degrees of freedom (denominator); DFn, degrees of freedom (numerator); *p*, probability value.

Supplementary Table 2. Reproducibility data for lesion volume at 24 hours, 7 days and 14 days after injury

		**25 mg/kg**		**40 mg/kg**		**60 mg/kg**	
		Lesion Vol.	Lesion Area (% Hemi.)	Lesion Vol.	Lesion Area (% Hemi.)	Lesion Vol.	Lesion Area (% Hemi.)
**24 hours**	**Mean**	3.74	8.15	5.33	8.36	11.15	11.76
	**SD**	1.16	2.20	1.07	1.81	1.27	1.85
	**CV (%)**	31%	27%	20%	22%	11%	16%
**7 days**	**Mean**	0.71		1.79		2.69	
	**SD**	0.32		0.49		0.69	
	**CV (%)**	45%		29%		26%	
**14 days**	**Mean**	0.05		0.79		2.02	
	**SD**	0.01		0.94		2.05	
	**CV (%)**	26%		119%		101%	

Data are presented as mean (mm^3) ± SD (*n* = 4/dose). Abbreviations: CV, coefficient of variation; Hemi., hemisphere; SD, standard deviation; Vol., volume.

Supplementary Table 3. Results of the statistical analysis for cortical atrophy and ventricle enlargement

Figure	Test name	Main effects			Sidak's multiple comparison			
		Interaction F (DFn, DFd), p value	Rose Bengal dose, F (DFn, DFd), p value	Time post-injury, F (DFn, DFd), p value	Time post-injury	Rose Bengal Dose comparisons	95% CI	p value
**Figure 2D,** Cortical atrophy	Two-way ANOVA	F (6, 36) = 1.811, p = 0.1245	F (3, 36) = 26.74, ***p < 0.0001***	F (2, 36) = 9.881, ***p = 0.0004***	24 h	Sham vs 25 mg/kg	-9.479 to 0.8295	0.1420
						Sham vs 40 mg/kg	-9.677 to 0.6322	0.1122
						Sham vs 60 mg/kg	-11.57 to -1.263	***0.0083***
						25 mg/kg vs 40 mg/kg	-5.352 to 4.957	>0.9999
						25mg/kg vs 60 mg/kg	-7.247 to 3.062	0.8435
						40 mg/kg vs 60 mg/kg	-7.050 to 3.259	0.8947
					7 d	Sham vs 25 mg/kg	-14.96 to -4.656	***<0.0001***
						Sham vs 40 mg/kg	-12.81 to -2.504	***0.0012***
						Sham vs 60 mg/kg	-18.59 to -8.276	***<0.0001***
						25 mg/kg vs 40 mg/kg	-3.002 to 7.306	0.8260
						25 mg/kg vs 60 mg/kg	-8.775 to 1.534	0.3030
						40 mg/kg vs 60 mg/kg	-10.93 to -0.6178	***0.0213***
					14 d	Sham vs 25 mg/kg	-9.663 to 0.6457	0.1141
						Sham vs 40 mg/kg	-11.91 to -1.599	***0.0050***
						Sham vs 60 mg/kg	-13.20 to -2.892	***0.0007***
						25 mg/kg vs 40 mg/kg	-7.399 to 2.910	0.7971
						25 mg/kg vs 60 mg/kg	-8.692 to 1.617	0.3278
						40 mg/kg vs 60 mg/kg	-6.448 to 3.861	0.9823
**Figure 2E,**	Two-way	F (6, 36) =	F (3, 36) =	F (2, 36) =	24 h	Sham vs. 25 mg/kg	-22.72 to -2.906	***0.0057***

Ventricle enlargement	ANOVA	1.087, p = 0.3884	12.22, ***p < 0.0001***	0.5672, p = 0.5721		Sham vs 40 mg/kg	-18.84 to 0.9762	0.0963
						Sham vs 60 mg/kg	-22.53 to -2.711	***0.0066***
						25 mg/kg vs 40 mg/kg	-6.026 to 13.79	0.8637
						25 mg/kg vs 60 mg/kg	-9.714 to 10.10	>0.9999
						40 mg/kg vs 60 mg/kg	-13.60 to 6.221	0.8894
					7 d	Sham vs 25 mg/kg	-15.00 to 4.814	0.6511
						Sham vs 40 mg/kg	-13.57 to 6.246	0.8925
						Sham vs 60 mg/kg	-23.47 to -3.651	***0.0031***
						25 mg/kg vs 40 mg/kg	-8.476 to 11.34	0.9991
						25 mg/kg vs 60 mg/kg	-18.37 to 1.444	0.1294
						40 mg/kg vs 60 mg/kg	-19.81 to 0.011	0.0504
					14 d	Sham vs 25 mg/kg	-19.15 to 0.6687	0.0787
						Sham vs 40 mg/kg	-19.44 to 0.3812	0.0640
						Sham vs 60 mg/kg	-19.46 to 0.3587	0.0987
						25 mg/kg vs 40 mg/kg	-10.20 to 9.621	>0.9999
						25 mg/kg vs 60 mg/kg	-10.22 to 9.599	>0.9999
						40 mg/kg vs 60 mg/kg	-9.931 to 9.886	>0.9999

Two-way ANOVA was used to analyse cortical atrophy and ventricle enlargement across three Rose Bengal doses and sham controls, with significance set at $p < 0.05$. Sidak's post hoc test was used for multiple comparisons. Bold and italicised values indicate statistically significant comparisons. Sample sizes: Sham = 4 rats, 25 mg/kg = 4 rats, 40 mg/kg = 4 rats, 60 mg/kg = 4 rats. **Abbreviations**: ANOVA, analysis of variance; CI, confidence interval; DFd, degrees of freedom (denominator); DFn, degrees of freedom (numerator); p, probability value.

Supplementary Table 4. Results of the statistical analysis for Cleaved Caspase-3 (CC3) cell counts in the infarct border and core.

Figure	Test name	Main effects			Sidak's multiple comparison			
		**Interaction F (DFn, DFd), p value**	**Rose Bengal dose, F (DFn, DFd), p value**	**Variables, F (DFn, DFd), p value**	**Time post-injury**	**Rose Bengal Dose comparisons**	**95% CI**	**p value**
**Figure 3A,** CC3 Infarct border	One-way ANOVA	–	F (3, 12) = 4.995, ***p = 0.0178***	–	24 h	Sham vs. 25 mg/kg	-126.4 to -10.60	***0.0197***
						Sham vs. 40 mg/kg	-125.4 to -9.596	***0.0215***
						Sham vs. 60 mg/kg	-119.2 to -3.346	***0.0373***
**Figure**	**Test name**	**Main effects**			**Sidak's multiple comparison**			
		**Interaction F (DFn, DFd), p value**	**Rose Bengal dose, F (DFn, DFd), p value**	**Time post-injury, F (DFn, DFd), p value**	**Time post-injury**	**Rose Bengal Dose comparisons**	**95% CI**	**p value**
**Figure 4A,** CC3 Infarct core	Two-way ANOVA	F (6, 36) = 5.376, ***p = 0.0005***	F (3, 36) = 16.22, ***p < 0.0001***	F (2, 36) = 53.94, ***p < 0.0001***	24 h	Sham vs. 25 mg/kg	-357.9 to 276.9	0.9996
						Sham vs. 40 mg/kg	-354.9 to 279.9	0.9997
						Sham vs. 60 mg/kg	-337.9 to 296.9	>0.9999
						25mg/kg vs. 40 mg/kg	-314.4 to 320.4	>0.9999
						25mg/kg vs. 60 mg/kg	-297.4 to 337.4	>0.9999
						40mg/kg vs. 60 mg/kg	-300.4 to 334.4	>0.9999
					7 d	Sham vs. 25 mg/kg	-650.9 to -16.09	***0.0351***
						Sham vs. 40 mg/kg	-651.9 to -17.09	***0.0343***
						Sham vs. 60 mg/kg	-650.7 to -15.84	***0.0353***
						25mg/kg vs. 40 mg/kg	-318.4 to 316.4	>0.9999
						25mg/kg vs. 60 mg/kg	-317.2 to 317.7	>0.9999
						40mg/kg vs. 60 mg/kg	-316.2 to 318.7	>0.9999
					14 d	Sham vs. 25 mg/kg	-1211 to -576.1	***<0.0001***

						Sham vs. 40 mg/kg	-987.9 to -353.1	***<0.0001***
						Sham vs. 60 mg/kg	-948.2 to -313.3	***<0.0001***
						25 mg/kg vs. 40 mg/kg	-94.41 to 540.4	0.3026
						25 mg/kg vs. 60 mg/kg	-54.66 to 580.2	0.1519
						40 mg/kg vs. 60 mg/kg	-277.7 to 357.2	0.9996

CC3-positive cell counts in the infarct border and core were analysed using one- or two-way ANOVA, with significance set at $p < 0.05$. Sidak's post hoc test was used for multiple comparisons. Bold and italicised values indicate statistically significant comparisons. Sample sizes: Sham = 4 rats, 25 mg/kg = 4 rats, 40 mg/kg = 4 rats, 60 mg/kg = 4 rats. **Abbreviations:** ANOVA, analysis of variance; CC3, cleaved caspase-3; CI, confidence interval; DFd, degrees of freedom (denominator); DFn, degrees of freedom (numerator); *p*, probability value.

Supplementary Table 5. Results of the statistical analysis for Iba1 and GFAP area coverage in the glial reactive zone

Figure	Test name	Main effects			Sidak's multiple comparison			
		Interaction F (DFn, DFd), p value	Rose Bengal dose, F (DFn, DFd), p value	Variables, F (DFn, DFd), p value	Time post-injury	Rose Bengal Dose comparisons	95% CI	p value
**Figure 3B,** Iba1 Infarct border	One-way ANOVA	–	F (3, 12) = 15.89, ***p = 0.0002***	–	24 h	Sham vs. 25 mg/kg	-13.51 to -5.226	***0.0001***
						Sham vs. 40 mg/kg	-11.84 to -3.553	***0.0007***
						Sham vs. 60 mg/kg	-11.96 to -3.673	***0.0006***
**Figure 3C,** GFAP Infarct border	One-way ANOVA	–	F (3, 12) = 36.89, ***p < 0.0001***	–	24 h	Sham vs. 25 mg/kg	-6.705 to -1.290	***0.0045***
						Sham vs. 40 mg/kg	-9.310 to -3.895	***< 0.0001***
						Sham vs. 60 mg/kg	-12.64 to -7.227	***< 0.0001***
**Supplementary Figure 4,** Iba1 Glial reactive zone width	One-way ANOVA	–	F (2, 9) = 3.085, p = 0.0954	–	24 h	25 mg/kg vs. 40 mg/kg	-28.08 to 70.40	0.5622
						25 mg/kg vs. 60 mg/kg	-7.394 to 91.08	0.1007
						40 mg/kg vs. 60 mg/kg	-28.56 to 69.92	0.5792

One-way ANOVA was used to assess the effects of dose on Iba1 and GFAP immunoreactivity in the infarct border and glial reactive zone at 24 h post-injury. Sidak's post hoc test was applied for multiple comparisons. Significance was set at $p < 0.05$. Bold and italicised values indicate statistically significant comparisons**.** Sample sizes: Sham = 4 rats, 25 mg/kg = 4 rats, 40 mg/kg = 4 rats, 60 mg/kg = 4 rats. **Abbreviations:** ANOVA, analysis of variance; CI, confidence interval; DFd, degrees of freedom (denominator); DFn, degrees of freedom (numerator); GFAP, glial fibrillary acidic protein; Iba1, ionised calcium-binding adaptor molecule 1; *p*, probability value.

Supplementary Table 6. Results of the statistical analysis for Iba1 and GFAP area coverage in the infarct core

Figure	Test name	Main effects			Sidak's multiple comparison			
		Interaction F (DFn, DFd), p value	Rose Bengal dose, F (DFn, DFd), p value	Time post-injury, F (DFn, DFd), p value	Time post-injury	Rose Bengal Dose comparisons	95% CI	p value
**Figure 4B,** Iba1 Infarct core	Two-way ANOVA	F (6, 36) = 10.25, ***p < 0.0001***	F (3, 36) = 31.78, ***p < 0.0001***	F (2, 36) = 70.81, ***p < 0.0001***	24 h	Sham vs. 25 mg/kg	-11.02 to 10.86	> 0.9999
						Sham vs. 40 mg/kg	-10.87 to 11.00	> 0.9999
						Sham vs. 60 mg/kg	-10.83 to 11.04	> 0.9999
						25 mg/kg vs. 40 mg/kg	-10.79 to 11.08	> 0.9999
						25 mg/kg vs. 60 mg/kg	-10.75 to 11.12	> 0.9999
						40 mg/kg vs. 60 mg/kg	-10.90 to 10.97	> 0.9999
					7 d	Sham vs. 25 mg/kg	-40.59 to -18.72	***< 0.0001***
						Sham vs. 40 mg/kg	-33.08 to -11.21	***< 0.0001***
						Sham vs. 60 mg/kg	-33.01 to -11.13	***< 0.0001***
						25 mg/kg vs. 40 mg/kg	-3.420 to 18.45	0.3264
						25 mg/kg vs. 60 mg/kg	-3.348 to 18.52	0.316
						40 mg/kg vs. 60 mg/kg	-10.86 to 11.01	> 0.9999
					14 d	Sham vs. 25 mg/kg	-37.47 to -15.60	***< 0.0001***
						Sham vs. 40 mg/kg	-30.14 to -8.265	***0.0001***
						Sham vs. 60 mg/kg	-47.53 to -25.66	***< 0.0001***
						25 mg/kg vs. 40 mg/kg	-3.603 to 18.27	0.3536
						25 mg/kg vs. 60 mg/kg	-21.00 to 0.8753	0.0855
						40 mg/kg vs. 60 mg/kg	-28.33 to -6.457	***0.0005***
**Figure 4C,** GFAP Infarct core	Two-way ANOVA	F (6, 36) = 4.997, ***p = 0.0008***	F (3, 36) = 19.82, ***p < 0.0001***	F (2, 36) = 41.89, ***p < 0.0001***	24 h	Sham vs. 25 mg/kg	-8.862 to 6.717	0.9823
						Sham vs. 40 mg/kg	-8.867 to 6.712	0.9821
						Sham vs. 60 mg/kg	-10.49 to 5.090	0.7871
						25 mg/kg vs. 40 mg/kg	-7.795 to 7.785	> 0.9999
						25 mg/kg vs. 60 mg/kg	-9.417 to 6.162	0.9424

							40 mg/kg vs. 60 mg/kg	-9.412 to 6.167	0.9429
					7 d		Sham vs. 25 mg/kg	-27.65 to -12.07	***< 0.0001***
							Sham vs. 40 mg/kg	-26.76 to -11.18	***< 0.0001***
							Sham vs. 60 mg/kg	-26.10 to -10.52	***< 0.0001***
							25 mg/kg vs. 40 mg/kg	-6.900 to 8.680	0.9867
							25 mg/kg vs. 60 mg/kg	-6.240 to 9.340	0.9497
							40 mg/kg vs. 60 mg/kg	-7.130 to 8.450	0.9957
					14 d		Sham vs. 25 mg/kg	-15.62 to -0.03796	***0.0485***
							Sham vs. 40 mg/kg	-19.31 to -3.733	***0.0017***
							Sham vs. 60 mg/kg	-20.34 to -4.760	***0.0006***
							25 mg/kg vs. 40 mg/kg	-11.48 to 4.095	0.5828
							25 mg/kg vs. 60 mg/kg	-12.51 to 3.067	0.3736
							40 mg/kg vs. 60 mg/kg	-8.817 to 6.762	0.9844

Significance set at p<0.05. The main effects and multiple comparisons using Sidak's post hoc test were reported. Bold + italicised values = statistically significant. Iba1 and GFAP; Rats: Sham controls = 4 pups, 25mg/kg= 4 pups, 40mg/kg= 4 pups, 60mg/kg= 4 pups. **Abbreviations**: ANOVA, analysis of variance; CI, confidence interval; DFd, degrees of freedom (denominator); DFn, degrees of freedom (numerator); GFAP, glial fibrillary acidic protein; Iba1, ionised calcium-binding adaptor molecule 1; *p*, probability value.

Supplementary Table 7. Summary of statistical analysis for behavioural tasks

Figure	Test name	Main effects			Sidak's multiple comparison			
		Interaction F (DFn, DFd), p value	Rose Bengal dose, F (DFn, DFd), p value	Variables, F (DFn, DFd), p value	Time post-injury	Rose Bengal Dose comparisons	95% CI	p value
**Figure 5B,** Wire hang	One-way ANOVA	–	F (3, 48) = 3.008, ***p* = 0.0393**	–	24 h	Sham vs. 25 mg/kg	0.2746 to 14.05	***0.0392***
						Sham vs. 40 mg/kg	-0.1718 to 13.24	0.0583
						Sham vs. 60 mg/kg	-3.694 to 10.08	0.5898
**Figure 5C,** Wire hang	One-way ANOVA	–	F (3, 33) = 2.470, *p* = 0.0792	–	7 d	Sham vs. 25 mg/kg	-0.6401 to 21.36	***0.0699***
						Sham vs. 40 mg/kg	-1.650 to 20.35	0.1154
						Sham vs. 60 mg/kg	-6.005 to 16.00	0.5975
**Figure 5D,** Cylinder rearing	Two-way ANOVA	F (6, 51) = 0.4369, *p* = 0.8508	F (3, 51) = 9.735, ***p* < 0.0001**	F (2, 51) = 1.171, *p* = 0.3182	12 d	**Left paw touches**		
						Sham vs. 25 mg/kg	2.098 to 15.90	***0.0067***
						Sham vs. 40 mg/kg	1.348 to 15.15	***0.0143***
						Sham vs. 60 mg/kg	1.348 to 15.15	***0.0143***
						**Right paw touches**		
						Sham vs. 25 mg/kg	0.2093 to 14.01	***0.0416***
						Sham vs. 40 mg/kg	-1.541 to 12.26	0.1715
						Sham vs. 60 mg/kg	-1.041 to 12.76	0.1181
						**Both paw touches**		
						Sham vs. 25 mg/kg	-1.402 to 12.40	0.155
						Sham vs. 40 mg/kg	-1.902 to 11.90	0.2204
						Sham vs. 60 mg/kg	-4.402 to 9.402	0.7563
**Figure 5E,** Cylinder rearing	One-way ANOVA	–	F (3, 17) = 5.190, ***p* = 0.0100**	–	12 d	Sham vs. 25 mg/kg	2.356 to 26.09	***0.0166***
						Sham vs. 40 mg/kg	1.441 to 25.17	***0.0256***
						Sham vs. 60 mg/kg	-0.9988 to 22.73	0.0782
**Figure 5F,**	One-way	_	F (3, 17) =	_	12 d	Sham vs. 25 mg/kg	-79.76 to -2.450	***0.0354***

Adhesive tape removal	ANOVA		2.947, p=0.0625			Sham vs. 40 mg/kg	-57.84 to 19.47	0.5002
						Sham vs. 60 mg/kg	-64.13 to 13.18	0.2689
**Figure 5G,** Adhesive tape removal	One-way ANOVA	–	F (3, 17) = 8.183, ***p = 0.0014***	–	12 d	Sham vs. 25 mg/kg	-1.260 to -0.2687	***0.0023***
						Sham vs. 40 mg/kg	-0.9571 to 0.033	0.0719
						Sham vs. 60 mg/kg	-1.217 to -0.2262	***0.0038***
**Supplementary Figure 6A,** Time spent in centre	One-way ANOVA	–	F (3, 46) = 0.279, p = 0.8401	–	24 h	Sham vs. 25 mg/kg	-39.05 to 37.65	> 0.9999
						Sham vs. 40 mg/kg	-50.13 to 26.56	0.8339
						Sham vs. 60 mg/kg	-48.44 to 30.16	0.9190
**Supplementary Figure 6A,** Time spent in border	One-way ANOVA	–	F (3, 46) = 0.276, p = 0.8422	–	24 h	Sham vs. 25 mg/kg	-38.14 to 39.01	> 0.9999
						Sham vs. 40 mg/kg	-26.81 to 50.34	0.8369
						Sham vs. 60 mg/kg	-30.66 to 48.41	0.9264
**Supplementary Figure 6A,** Line crossings	One-way ANOVA	–	F (3, 46) = 0.753, p = 0.5261	–	24 h	Sham vs. 25 mg/kg	-47.29 to 11.69	0.3675
						Sham vs. 40 mg/kg	-39.04 to 19.94	0.8114
						Sham vs. 60 mg/kg	-38.66 to 21.79	0.8695
**Supplementary Figure 6B,** Time spent in centre	One-way ANOVA	–	F (3, 32) = 0.182, p = 0.9072	–	7 d	Sham vs. 25 mg/kg	-12.71 to 11.79	0.9995
						Sham vs. 40 mg/kg	-9.837 to 14.67	0.9355
						Sham vs. 60 mg/kg	-9.712 to 14.79	0.9263
**Supplementary Figure 6B,** Time spent in border	One-way ANOVA	–	F (3, 32) = 0.143, p = 0.9333	–	7 d	Sham vs. 25 mg/kg	-11.48 to 13.73	0.9945
						Sham vs. 40 mg/kg	-14.36 to 10.86	0.9801
						Sham vs. 60 mg/kg	-14.48 to 10.73	0.9757
**Supplementary Figure 6B,** Line crossings	One-way ANOVA	–	F (3, 32) = 0.602, p = 0.6180	–	7 d	Sham vs. 25 mg/kg	-121.8 to 48.85	0.6417
						Sham vs. 40 mg/kg	-124.6 to 45.98	0.5849
						Sham vs. 60 mg/kg	-110.4 to 60.23	0.8463
**Supplementary Figure 6C,** Immobility time	One-way ANOVA	–	F (3, 44) = 1.250, p = 0.3032	–	24 h	Sham vs. 25 mg/kg	-25.79 to 4.872	0.2648
						Sham vs. 40 mg/kg	-24.44 to 5.476	0.3250
						Sham vs. 60 mg/kg	-21.10 to 10.44	0.7901
**Supplementary**	One-way	_	F (3, 46) =	_	24 h	Sham vs. 25 mg/kg	-4.906 to 10.21	0.7723

**Figure 6C,** Rearing time	ANOVA		0.412, p = 0.7447			Sham vs. 40 mg/kg	-4.573 to 10.54	0.7033
						Sham vs. 60 mg/kg	-5.435 to 10.05	0.8458
**Supplementary Figure 6D,** Immobility time	One-way ANOVA	–	F (3, 28) = 0.451, p = 0.7183	–	7 d	Sham vs. 25 mg/kg	-25.75 to 27.99	0.9994
						Sham vs. 40 mg/kg	-16.61 to 37.13	0.7133
						Sham vs. 60 mg/kg	-29.04 to 24.70	0.9958
**Supplementary Figure 6D,** Rearing time	One-way ANOVA	–	F (3, 32) = 0.329, p = 0.8037	–	7 d	Sham vs. 25 mg/kg	-13.34 to 17.01	0.9867
						Sham vs. 40 mg/kg	-19.34 to 11.01	0.8705
						Sham vs. 60 mg/kg	-17.59 to 12.76	0.9705

Behavioural data were analysed using one-way or two-way ANOVA, with significance set at $p<0.05$. The main effects and multiple comparisons using Sidak's post hoc test were reported. Bold + italicised values = statistically significant. 24h wire hang; Rats: Sham controls = 18 pups, 25mg/kg= 11 pups, 40mg/kg= 12 pups, 60mg/kg= 11 pups. 7d wire hang; Rats: Sham controls = 13 pups, 25mg/kg= 8 pups, 40mg/kg= 8 pups, 60mg/kg= 8 pups. Cylinder rearing and adhesive tape removal; Rats: Sham controls = 9 pups, 25mg/kg= 4 pups, 40mg/kg= 4 pups, 60mg/kg= 4 pups. Open field: Data from 24 hours (Supplementary Figure 6A, 6C) and 7 days post-injury (Supplementary Figure 6B, 6D). At 24h post-injury: Sham controls = 15 pups, 25mg/kg= 12 pups, 40mg/kg= 12 pups, 60mg/kg= 11 pups. At 7d post-injury: Sham controls = 12 pups, 25mg/kg= 8 pups, 40mg/kg= 8 pups, 60mg/kg= 8 pups.

Supplementary Table 8. Exploratory correlations between behavioural outcomes and lesion volumes.

	**Pearson r**	**R^2**	***p* value**
**Wire Hang Time at 24 hours vs. lesion volume at 24 hours**			
**25 mg/kg RB**	–0.526	0.277	0.236
**40 mg/kg RB**	–0.718	0.515	0.245
**60 mg/kg RB**	0.029	0.0008	0.490
**25 and 40 mg/kg**	–0.633	0.400	*0.063*
**25 and 40 and 60 mg/kg**	0.330	0.1091	0.175
**Wire Hang Time at 24 hours vs lesion volume at 7 days**			
**25 mg/kg RB**	–0.813	0.661	*0.093*
**40 mg/kg RB**	–0.995	0.991	*0.002*
**60 mg/kg RB**	0.326	0.106	0.336
**12-day contralateral/ipsilateral paw use versus lesion volume at 14 days**			
**25 mg/kg RB**	0.946	0.895	*0.026*
**40 mg/kg RB**	–0.167	0.028	0.416
**60 mg/kg RB**	–0.161	0.026	0.419

Abbreviations: RB: Rose Bengal

Supplementary Table 9. Summary of sex-specific statistical analysis for the 25 mg/kg Rose Bengal dose

This table presents the results of two-way ANOVA analyses assessing the main and interaction effects of sex, injury, and time post-injury on histological (infarct volume, cleaved caspase-3, GFAP, Iba1) and behavioural (wire hang) outcomes following a 25 mg/kg Rose Bengal–induced focal ischemic injury. Sidak's multiple comparisons were applied to explore sex- and injury-related differences across time points (24 h, 7 d, 14 d). Reported values include F(DFn, DFd), corresponding p-values, 95% confidence intervals (CI), and post hoc pairwise comparisons.

Figure	***Test name***	**Main effects**			**Sidak's multiple comparison**			
		Interaction F (DFn, DFd), p value	***Sex, F (DFn, DFd), p value***	***Time post-injury, F (DFn, DFd), p value***	***Time post-injury***	***Sex/injury comparisons***	***95% CI***	***p value***
**Figure 6A,** Infarct volume	Two-way ANOVA	F (2, 18) = 0.9857, p = 0.3924	F (1, 18) = 1.034, p = 0.3227	F (2, 18) = 38.46, ***px < 0.0001***	24h	Males - Females	-0.563 to 2.696	0.2766
					7d		-1.529 to 1.731	0.9979
					14d		-1.706 to 1.554	0.9991
**Figure**	**Test name**	**Interaction F (DFn, DFd), p value**	**Sex, F (DFn, DFd), p value**	**Injury, F (DFn, DFd), p value**	**Injury/Sex**	**Sex/injury comparisons**	**95% CI**	**p value**
**Figure 6B,** Wire hang 24 h	Two-way ANOVA	F (1, 39) = 0.1337, p = 0.7166	F (1, 39) = 0.6780, p = 0.4153	F (1, 39) = 14.54, ***p = 0.0005***	Sham	Males - Females	-3.831 to 8.564	0.6155
					Injury		-5.967 to 7.789	0.9422
					Males	Sham - Injury	1.623 to 15.01	***0.0125***
					Fem.		0.4661 to 13.26	***0.0336***
**Figure 6B,** Wire hang 7 d	Two-way ANOVA	F (1, 32) = 0.3341, *p* = 0.5673	F (1, 32) = 0.02398, *p* = 0.8779	F (1, 32) = 4.619, ***p = 0.0393***	Sham	Males - Females	-11.65 to 8.809	0.9357
					Injury		-9.545 to 14.47	0.8657
					Males	Sham - Injury	-6.411 to 16.98	0.5060
					Fem.		-1.422 to 19.76	0.0986
**Figure 6C,**	Two-way	F (1, 12) =	F (1, 12) =	F (1, 12) =	Sham	Males - Females	-56.26 to 49.76	0.9852

CC3 Infarct border 24 h	ANOVA	0.03197, *p* = 0.8611	0.001812, *p* = 0.9667	14.22, ***p* = *0.0027***	Injury		-51.01 to 55.01	0.9944
					Males	Sham - Injury	-111 to -4.988	***0.0322***
					Fem.		-105.8 to 0.262	0.0512
**Figure 6D,** Iba1 Infarct border 24 h	Two-way ANOVA	F (1, 12) = 5.866e, *p* = 0.9940	F (1, 12) = 0.01790, *p* = 0.8958	F (1, 12) = 148.4, *p* < 0.0001	Sham	Males - Females	-3.123 to 2.888	0.9939
					Injury		-3.110 to 2.900	0.9952
					Males	Sham - Injury	-13.15 to -7.14	***< 0.0001***
					Fem.		-13.14 to -7.12	***< 0.0001***
**Figure 6E,** GFAP Infarct border 24 h	Two-way ANOVA	F (1, 12) = 0.1033, *p* = 0.7535	F (1, 12) = 0.1303, *p* = 0.7243	F (1, 12) = 221.5, ***p* < *0.0001***	Sham	Males - Females	-1.125 to 1.150	0.9995
					Injury		-0.9226 to 1.35	0.8690
					Males	Sham - Injury	-5.928 to -3.65	***< 0.0001***
					Fem.		-5.725 to -3.45	***< 0.0001***
**Figure**	**Test name**	**Interaction F (DFn, DFd), *p* value**	**Injury/Sex, F (DFn, DFd), *p* value**	**Time post-injury, F (DFn, DFd), *p* value**	**Time post-injury**	**Sex/injury comparisons**	**95% CI**	**p value**
**Figure 6D,** Iba1 Infarct core	Two-way ANOVA	F (6, 36) = 33.66, ***p* < *0.0001***	F (3, 36) = 131.1, ***p* < *0.0001***	F (2, 36) = 103.8, ***p* < *0.0001***	24 h	Sham Males vs. Injury Males	-6.861 to 7.406	> 0.9999
						Sham Males vs. Sham Females	-7.251 to 7.016	> 0.9999
						Sham Males vs. Injury Females	-7.479 to 6.789	> 0.9999
						Injury Males vs. Sham Females	-7.524 to 6.743	> 0.9999
						Injury Males vs. Injury Females	-7.751 to 6.516	> 0.9999
						Sham Females vs. Injury Females	-7.361 to 6.906	> 0.9999
					7 d	Sham Males vs. Injury Males	-35.41 to -21.1	***< 0.0001***
						Sham Males vs. Sham Females	-7.651 to 6.616	> 0.9999
						Sham Males vs. Injury Females	-42.42 to -28.1	***< 0.0001***
						Injury Males vs. Sham Females	20.62 to 34.89	***< 0.0001***
						Injury Males vs. Injury Females	-14.14 to 0.123	0.0563
						Sham Females vs. Injury Females	-41.90 to -27.6	***< 0.0001***
					14 d	Sham Males vs. Injury Males	-38.16 to -23.8	***< 0.0001***

						Sham Males vs. Sham Females	-6.741 to 7.526	> 0.9999
						Sham Males vs. Injury Females	-36.88 to -22.6	***< 0.0001***
						Injury Males vs. Sham Females	24.28 to 38.55	***< 0.0001***
						Injury Males vs. Injury Females	-5.854 to 8.414	0.997
						Sham Females vs. Injury Females	-37.27 to -23.0	***< 0.0001***
**Figure 6E,** GFAP Infarct core	Two-way ANOVA	F (6, 36) = 15.42, ***p < 0.0001***	F (3, 36) = 56.22, ***p < 0.0001***	F (2, 36) = 50.11, ***p < 0.0001***	24 h	Sham Males vs. Injury Males	-6.783 to 4.543	0.9949
						Sham Males vs. Sham Females	-5.651 to 5.676	> 0.9999
						Sham Males vs. Injury Females	-6.543 to 4.783	0.9987
						Injury Males vs. Sham Females	-4.531 to 6.796	0.9946
						Injury Males vs. Injury Females	-5.423 to 5.903	> 0.9999
						Sham Females vs. Injury Females	-6.556 to 4.771	0.9985
					7 d	Sham Males vs. Injury Males	-26.18 to -14.8	***< 0.0001***
						Sham Males vs. Sham Females	-5.634 to 5.692	>0.9999
						Sham Males vs. Injury Females	-26.20 to -14.8	***< 0.0001***
						Injury Males vs. Sham Females	14.88 to 26.21	***< 0.0001***
						Injury Males vs. Injury Females	-5.681 to 5.646	> 0.9999
						Sham Females vs. Injury Females	-26.23 to -14.9	***< 0.0001***
					14 d	Sham Males vs. Injury Males	-16.30 to -4.96	***< 0.0001***
						Sham Males vs. Sham Females	-6.358 to 4.968	0.9997
						Sham Males vs. Injury Females	-17.33 to -6.00	***< 0.0001***
						Injury Males vs. Sham Females	4.274 to 15.60	***0.0001***
						Injury Males vs. Injury Females	-6.701 to 4.626	0.9967
						Sham Females vs. Injury Females	-16.64 to -5.31	***< 0.0001***
**Figure 6C,** CC3 Infarct core	Two-way ANOVA	F (6, 36) = 26.47, ***p < 0.0001***	F (3, 36) = 65.83, ***p < 0.0001***	F (2, 36) = 90.40, ***p < 0.0001***	24 h	Sham Males vs. Injury Males	-226.8 to 188.8	> 0.9999
						Sham Males vs. Sham Females	-211.0 to 204.5	> 0.9999
						Sham Males vs. Injury Females	-217 to 198.5	> 0.9999
						Injury Males vs. Sham Females	-192 to 223.5	> 0.9999

						Injury Males vs. Injury Females	-198 to 217.5	> 0.9999
						Sham Females vs. Injury Females	-213.8 to 201.8	> 0.9999
					7 d	Sham Males vs. Injury Males	-595.8 to -180.	***< 0.0001***
						Sham Males vs. Sham Females	-213.5 to 202	> 0.9999
						Sham Males vs. Injury Females	-487.5 to -71.9	***0.0037***
						Injury Males vs. Sham Females	174.5 to 590	***< 0.0001***
						Injury Males vs. Injury Females	-99.55 to 316	0.6378
						Sham Females vs. Injury Females	-481.8 to -66.2	***0.0047***
					14 d	Sham Males vs. Injury Males	-1155 to - 739.7	***< 0.0001***
						Sham Males vs. Sham Females	-178.5 to 237	0.9992
						Sham Males vs. Injury Females	-1102 to - 686.7	***< 0.0001***
						Injury Males vs. Sham Females	769 to 1185	***< 0.0001***
						Injury Males vs. Injury Females	-154.8 to 260.8	0.9807
						Sham Females vs. Injury Females	-1132 to -716	***< 0.0001***

Figure	Test name	Interaction F (DFn, DFd), p value	Sex, F (DFn, DFd), p value	Injury, F (DFn, DFd), p value	Injury/Sex	Sex/injury comparisons	95% CI	p value
**Supplementary Figure 7A,** Time spent in centre 24 h	Two-way ANOVA	F (1, 36) = 0.398, p = 0.5317	F (1, 36) = 0.454, p = 0.5047	F (1, 36) = 2.072, p = 0.1586	Sham	Males - Females	-34.08 to 34.97	0.9994
					Injury		-20.87 to 48.18	0.5931
					Males	Sham - Injury	-57.88 to 14.54	0.3130
					Fem.		-41.21 to 24.30	0.7981
**Supplementary Figure 7A,** Time spent in border 24 h	Two-way ANOVA	F (1, 36) = 0.419, p = 0.5213	F (1, 36) = 0.429, p = 0.5164	F (1, 36) = 2.015, p = 0.1643	Sham	Males - Females	-34.67 to 34.51	> 0.9999
					Injury		-48.24 to 20.93	0.5942
					Males	Sham - Injury	-14.61 to 57.94	0.3143
					Fem.		-24.72 to 40.90	0.8139
**Supplementary**	Two-way	F (1, 36) =	F (1, 36) =	F (1, 36) =	Sham	Males - Females	-52.47 to 17.54	0.4404

**Figure 7A,** Line crossings 24 h	ANOVA	0.002, p = 0.9608	2.551, p = 0.1190	0.934, p = 0.3402	Injury		-51.42 to 18.59	0.4832
					Males	Sham - Injury	-47.49 to 25.93	0.7477
					Fem.		-42.93 to 23.48	0.7486
**Supplementary Figure 7B,** Time spent in centre 7 d	Two-way ANOVA	F (1, 30) = 0.173, p = 0.6800	F (1, 30) = 0.018, p = 0.8918	F (1, 30) = 5.184, p = 0.0301	Sham	Males - Females	-27.72 to 23.05	0.9712
					Injury		-25.41 to 34.66	0.9214
					Males	Sham - Injury	-52.54 to 7.540	0.1684
					Fem.		-40.93 to 9.847	0.2943
**Supplementary Figure 7B,** Time spent in border 7 d	Two-way ANOVA	F (1, 30) = 0.022, p = 0.8817	F (1, 30) = 0.019, p = 0.8906	F (1, 30) = 4.378, p = 0.0450	Sham	Males - Females	-26.49 to 26.68	> 0.9999
					Injury		-31.34 to 26.52	0.9762
					Males	Sham - Injury	-11.16 to 48.59	0.2788
					Fem.		-9.304 to 41.72	0.2694
**Supplementary Figure 7B,** Line crossings 7 d	Two-way ANOVA	F (1, 30) = 0.038, p = 0.8453	F (1, 30) = 0.072, p = 0.7895	F (1, 30) = 0.017, p = 0.8966	Sham	Males - Females	-97.07 to 72.28	0.9286
					Injury		-94.07 to 90.21	0.9985
					Males	Sham - Injury	-103.9 to 86.45	0.9714
					Fem.		-79.51 to 83.01	0.9984
**Supplementary Figure 7C,** Immobility time 24 h	Two-way ANOVA	F (1, 36) = 0.561, p = 0.4584	F (1, 36) = 0.002, p = 0.9644	F (1, 36) = 0.007, p = 0.9338	Sham	Males - Females	-14.37 to 23.48	0.8217
					Injury		-22.96 to 14.88	0.8566
					Males	Sham - Injury	-15.07 to 24.62	0.8217
					Fem.		-21.77 to 14.13	0.8576
**Supplementary Figure 7C,** Rearing time 24 h	Two-way ANOVA	F (1, 36) = 0.016, p = 0.8998	F (1, 36) = 0.430, p = 0.5159	F (1, 36) = 2.214, p = 0.1454	Sham	Males - Females	-5.715 to 9.271	0.8263
					Injury		-6.291 to 8.695	0.9161
					Males	Sham - Injury	-4.192 to 11.53	0.4866
					Fem.		-4.018 to 10.20	0.5336
**Supplementary Figure 7D,** Immobility time 7 d	Two-way ANOVA	F (1, 30) = 2.024, p = 0.1652	F (1, 30) = 0.3282, p = 0.5710	F (1, 30) = 1.382, p = 0.2490	Sham	Males - Females	-16.95 to 29.29	0.7836
					Injury		-39.64 to 10.68	0.3367
					Males	Sham - Injury	-7.127 to 44.84	0.1862
					Fem.		-23.98 to 20.40	0.9777

**Supplementary Figure 7D,** Rearing time 7 d	Two-way ANOVA	F (1, 30) = 0.245, p = 0.6238	F (1, 30) = 0.048, p = 0.8279	F (1, 30) = 1.308, p = 0.2617	Sham	Males - Females	-11.41 to 13.57	0.9743
					Injury		-16.40 to 10.79	0.8637
					Males	Sham - Injury	-7.610 to 20.47	0.4954
					Fem.		-9.446 to 14.53	0.8566

Significance set at $p < 0.05$. The main effects and multiple comparisons using Sidak's post hoc test were reported. Bold + italicised values = statistically significant. Infarct volume, Iba1 and GFAP area coverage (Figure 8A, 8D–G): 4 rat pups per group. 24h wire hang; Rats: Sham controls- Males = 10 pups, Females = 14 pups; Injury - Males = 10 pups, Females = 9 pups. 7d wire hang; Rats: Sham controls - Males = 9 pups, Females = 12 pups; Injury- Males = 7 pups, Females = 8 pups. Open field: Data from 24 hours (Supplementary Figure 7A, 7C) and 7 days (Supplementary Figure 7B, 7D) in males and females. At 24 hours post-injury, Sham controls- Males = 9 pups, Females = 11 pups; Injury - Males = 9 pups, Females = 11 pups. At 7 days post-injury, Sham controls- Males = 8 pups, Females = 12 pups; Injury - Males = 6 pups, Females = 8 pups. **Abbreviations:** CC3, cleaved caspase-3; CI, confidence interval; DFd, degrees of freedom (denominator); DFn, degrees of freedom (numerator); GFAP, glial fibrillary acidic protein; Iba1, ionised calcium-binding adaptor molecule 1; P, postnatal day; *p*, probability value.

Supplementary Table 10. Power calculation and effect size for lesion volume and behavioural outcomes by sex.

	**Males Mean ± SD**	**Females Mean ± SD**	**Cohen's d**	**Interpretation**	***N* per group for $p < 0.05$**
Lesion volume analyses over time (male vs female)					
**24 h**	4.35 ± 1.85	3.28 ± 0.41	0.75	Large	29
**7 d**	0.93 ± 0.66	0.83 ± 0.29	0.22	Small	385
**14 d**	0.16 ± 0.13	0.24 ± 0.18	0.44	Medium	80
Wire hang at 24 hours (sham vs injured by sex)					
**Sham**	17.05 ± 7.39	15.31 ± 7.96	0.23	Small	297
**Injury**	8.43 ± 2.71	7.70 ± 2.85	0.26	Small	232
Wire hang at 7 days (sham vs injured by sex)					
**Sham**	18.58 ± 9.28	20.20 ± 13.11	0.14	Small	801
**Injury**	14.77 ± 8.58	12.74 ± 3.99	0.30	Small	174

Abbreviations: *d* (Cohen's d), effect size; *d,* days; *h*, hours; *N*, total sample size; SD, standard deviation; *vs*, versus.

Supplementary Table 11. Sex distribution of rat pups used in figures 2–4 across experimental groups

Figure	Time post-injury	Stroke doses	Male (n)	Female (n)
**Figure 2B**	24 h	25 mg/kg	2	2
		40 mg/kg	2	2
		60 mg/kg	3	1
	7 d	25 mg/kg	2	2
		40 mg/kg	2	2
		60 mg/kg	1	3
	14 d	25 mg/kg	2	2
		40 mg/kg	2	2
		60 mg/kg	2	2
**Figure 3A–C**	24 h	Sham	2	2
		25 mg/kg	2	2
		40 mg/kg	2	2
		60 mg/kg	3	1
**Figure 2D–E, 4A-C**	24 h	Sham	2	2
		25 mg/kg	2	2
		40 mg/kg	2	2
		60 mg/kg	3	1
	7 d	Sham	1	3
		25 mg/kg	2	2
		40 mg/kg	2	2
		60 mg/kg	1	3
	14 d	Sham	2	2
		25 mg/kg	2	2
		40 mg/kg	2	2
		60 mg/kg	2	2

Abbreviations: h, hour; d, day; n, number.
